# Extracellular Vesicle‐Based mRNA Therapeutics and Vaccines

**DOI:** 10.1002/EXP.20240109

**Published:** 2025-08-07

**Authors:** Qi Li, Haonan Xing, Abid Naeem, Kaiyue Zhang, Aiping Zheng, Yuanyu Huang, Mei Lu

**Affiliations:** ^1^ School of Life Science, School of Interdisciplinary Science, Aerospace Center Hospital, Key Laboratory of Molecular Medicine and Biotherapy, Key Laboratory of Medical Molecule Science and Pharmaceutics Engineering Beijing Institute of Technology Beijing China; ^2^ School of Medical Engineering School of Interdisciplinary Science Affiliated Zhuhai People's Hospital, Beijing Institute of Technology Zhuhai China; ^3^ Advanced Technology Research Institute Beijing Institute of Technology Jinan China; ^4^ Chinese Academy of Sciences (CAS) Key Laboratory for Biomedical Effects of Nanomaterials and Nanosafety, CAS Center for Excellence in Nanoscience National Center for Nanoscience and Technology of China Beijing China; ^5^ Department of Biomedical Engineering Columbia University New York USA; ^6^ State Key Laboratory of Toxicology and Medical Countermeasures Beijing Institute of Pharmacology and Toxicology Beijing China

**Keywords:** extracellular vesicles, messenger RNAs, delivery, packaging strategies, vaccines

## Abstract

Messenger RNA (mRNA) therapeutics and vaccines have recently gained particular prominence following the COVID‐19 epidemic. However, clinical translation of mRNAs is critically dependent on efficient and safe delivery in vivo. Currently, a plethora of mRNA delivery technology platforms (such as lipid nanoparticles) have been developed and have achieved stunning success. Nevertheless, many challenges remain to be overcome, including immunogenicity and toxicities, excessive liver accumulation, limited endosomal escape ability, low tissue bioavailability, poor mucosal immunity, and the need for cold chain storage. In recent years, extracellular vesicles (EVs) have emerged as an attractive mRNA delivery platform due to their favorable properties, such as low immunogenicity, natural capability to deliver RNAs, intrinsic targeting capacity, and the ability to negotiate with physiological barriers. In this review, we discuss the latest efforts to harness EVs for mRNA delivery and elaborate the behind mechanisms, aiming to offering insights into the rational design of effective and safe EV‐based mRNA therapeutics and vaccines for biomedical applications. Additionally, we provide an overview of EV biogenesis, composition, cellular internalization, and their superiorities and challenges for mRNA delivery, with special emphasis on the state‐of‐the‐art methodologies for packaging EVs with mRNAs.

## Graphical abstract

This review provides a comprehensive analysis of recent advancements in extracellular vesicle (EV)‐based messenger RNA (mRNA) applications for therapeutics and vaccines. It systematically outlines strategies for mRNA encapsulation within EVs and elucidates the challenges associated with this technology, offering insights into future research directions in the field.

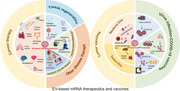

## 1 Introduction

Since the outbreak of COVID‐19, messenger RNAs (mRNAs) have emerged as a promising vaccine tool, providing potent immune responses that prevent the spread of pathogens. Compared with conventional therapy modalities, mRNA offers a swift and highly personalized treatment, since the fabrication could be proceeded rapidly, upon identification of the target and mRNA can be tailored according to the patient's genetic information. In contrast to DNA technology, mRNA functions in the cytoplasm instead of the nucleus, thus mitigating the risk of insertional mutagenesis, gene mutations and oncogenesis. Therefore, mRNA therapeutics and vaccines have gained significant attention [1]. However, the immune system could recognize RNA molecules entering the systemic circulation as foreign substances and swiftly clear them. Besides, these circulating RNAs may also be degraded by RNases [2]. These factors may result in limited therapeutic efficacy of mRNAs without a delivery system capable of enhancing their in vivo stability. In addition, how to facilitate the cytosolic delivery of mRNAs to receptor cells is another obstacle that inhibits the translation of mRNAs into functional proteins. Despite reaching the surface of cells, mRNAs cannot enter the cells due to their large size and charge repulsion [3]. Furthermore, efficient endosomal escape and cytoplasmic release are critical issues for mRNA translation and exerting therapeutic effects against diseases [4].

Delivery platforms that can effectively address the aforementioned challenges are urgently needed for mRNA delivery. Amongst various types of current mRNA delivery technologies, lipid nanoparticles (LNPs) are the most popular platforms for both therapeutic and vaccination purposes [5, 6, 7, 8, 9]. LNPs could protect mRNA molecules from nuclease degradation and promote their cellular internalization. However, challenges such as high immunogenicity and toxicity, inefficient extrahepatic delivery, limited endosomal escape efficiency, low pulmonary bioavailability, poor mucosal immunity, and requirement of cold chain greatly impede the clinical application of these vehicles and other nanoparticles [10]. Therefore, the quest for new vectors capable of overcoming these challenges is essential to advance mRNAs as a paradigm in the realm of therapeutics and vaccines.

Extracellular vesicles (EVs) are membrane‐bound particles secreted by cells into the extracellular space [11, 12, 13, 14, 15, 16]. These vesicles possess intrinsic immune privilege to evade the immune system, thereby being relatively stable in blood circulation [17]. In addition, EVs can cross natural physiological barriers like the blood‐brain barrier (BBB) and have their own targeting ability. For example, native macrophage‐derived EVs could cross the BBB by carrying intercellular adhesion molecule 1 (ICA‐1) and lymphocyte function‐associated antigen 1(LFA‐1) [18]. EVs derived from specific cell types, such as immune cells and tumor cells, possess homing properties, thus endowing them with efficient lymphatic accumulation and tumor‐targeting ability [19, 20]. Moreover, lyophilized EVs have been shown to be stable after being stored at room temperature for months, thus circumventing the requirement of the cold chain [21]. After inhalation of EV dry powder, effective pulmonary bioavailability and mucosal immunity could induce stronger immunity, resulting in rapid virus clearance [21]. Last, it is noteworthy that EVs are less toxic and less immunogenic than exogenous nanoparticles, which greatly supports multiple repeated drug dosing. These superior characteristics of EVs over LNPs make them an excellent delivery platform for mRNAs. Recently, EV‐based mRNA delivery systems have attracted considerable attention for the treatment of various diseases such as cancers, infectious diseases, tissue injuries, neurodegenerative diseases, leukemia, and obesity [22, 23, 24]. For example, mRNA encoding SARS‐CoV‐2 spike protein was loaded into EVs, and it induced strong immunity against multiple SARS‐CoV‐2 variants after immunization in mice [25]. Also, EVs could be used to deliver anti‐tumorigenic mRNAs encoding PTEN (phosphatase and tensin homologue) or CD‐UPRT (cytosine deaminase fused to uracil phosphoribosyltransferase) to suppress tumor proliferation and extend survival time in mice. In addition, Red blood cell (RBC) EVs loaded with CRISPR‐Cas9 mRNA were used to downregulate the expression of miR‐125a and miR‐125b in leukemia cells [22, 24, 26, 27, 28]. These results collectively demonstrate the promise of EVs to serve as an efficient mRNA delivery platform.

In this review, we outline EV composition, biogenesis, cellular internalization, and their superiorities and challenges for mRNA delivery with special emphasis on the state‐of‐the‐art methodologies for the efficient packaging of mRNAs. Additionally, we summarize the state‐of‐the‐art development of EV‐based mRNA therapeutics and vaccines and their biomedical applications in various fields, including cancers, tissue injuries, neurodegenerative diseases, infections, hematological diseases, vascular diseases, and obesity. We will also explore future research directions, focusing on the challenges that limit the efficient clinical translation of EV‐based mRNA therapeutics and vaccines. Through these efforts, we hope that the research discussed in this review will achieve significant breakthroughs in the future, advancing this field further.

## 2 EV Composition, Biogenesis, and Cellular Internalization

### 2.1 EV Composition

**FIGURE 1:**
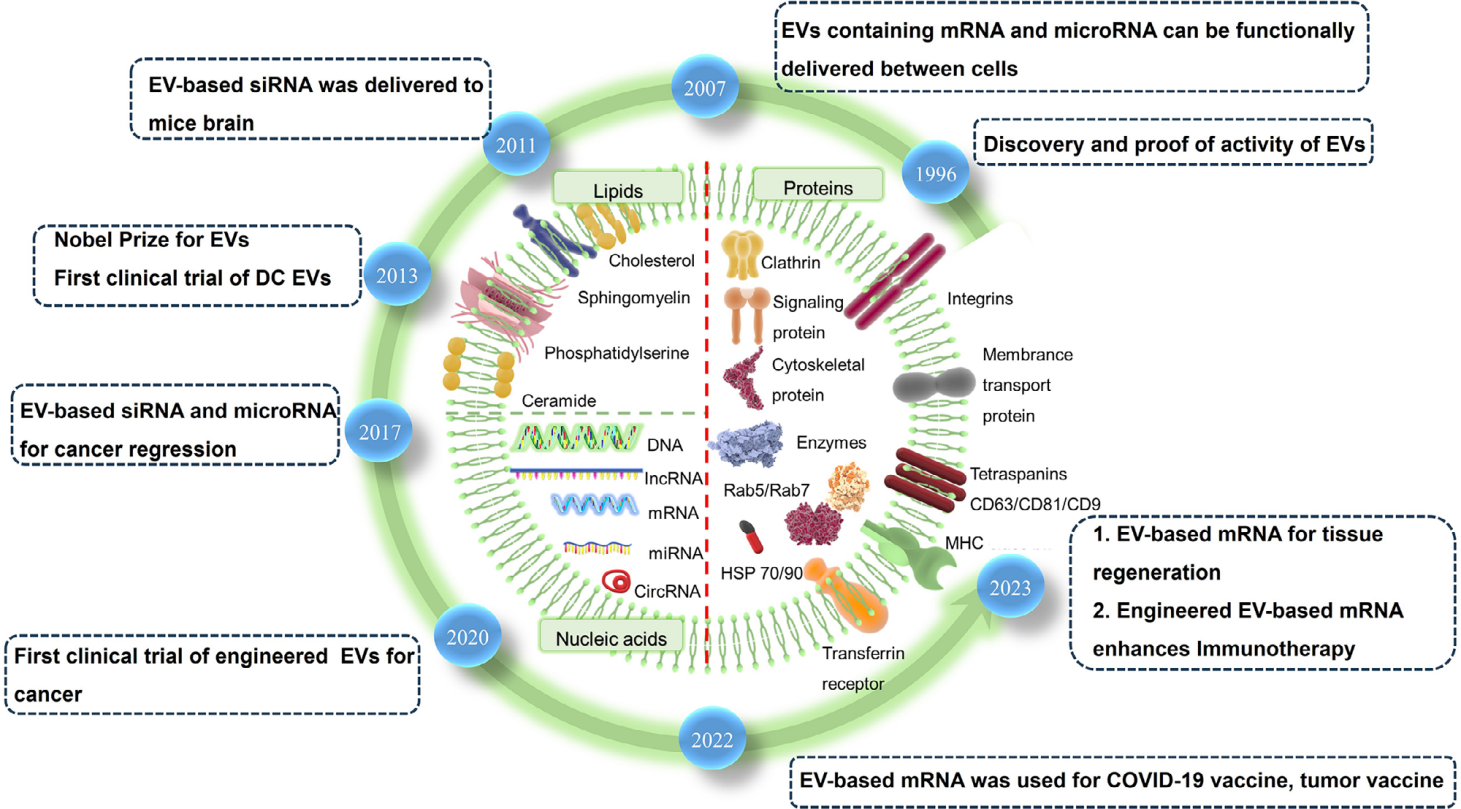
Historical overview of the development process of EV‐based mRNA and the components of EVs. EVs, first identified in 1940, play a crucial role in intercellular communication, a concept established in the 1990s. The transfer of mRNA and microRNAs via EVs, discovered in the 2000s, enables gene exchange between cells. This led to the development of EV‐based therapies utilizing siRNA and microRNA for cancer treatment, which have entered clinical trials. One of the early clinical trials involving engineered EVs for cancer immunotherapy was conducted in 2020 [35]. During the SARS‐CoV‐2 pandemic, EVs were successfully used for mRNA delivery in vaccine development due to their safety and rapid efficacy. Additionally, EV‐based mRNA was applied in tumor vaccines in 2023, highlighting ongoing advancements in this field. EVs are nanoscale vesicles secreted by various cells, and their composition is complex, primarily comprising phospholipids, nucleic acids, and proteins. Phospholipids enable their stability in body fluids and protect substances inside the EVs from being degraded. Nucleic acids, such as DNAs, mRNAs, microRNAs, and other non‐coding RNAs, can be carried and transferred to other cells by EVs, thereby influencing gene expression and cell behavior in the recipient cells. Proteins are not only involved in the formation and release of the EVs but also may play roles in signal transduction, cell adhesion, and other biological processes. HSP, heat shock protein; MHC, major histocompatibility complex.

EVs were first identified in 1940 and have since been recognized for their pivotal role in intercellular communication, a concept solidified during the 1990s. Gene exchange between cells is achieved through the transfer of mRNA and microRNAs via EVs, a phenomenon which first came to light in the 2000s [29]. Subsequently, EV‐based therapies utilizing siRNA and microRNA emerged as promising approaches for cancer regression, leading to their initiation in clinical trials [30]. Amidst the global SARS‐CoV‐2 pandemic, EVs have been effectively harnessed for delivering mRNA in SARS‐CoV‐2 vaccine development, owing to the favorable safety profile and rapid efficacy of mRNA [21]. Additionally, EV‐based mRNA has been applied in tumor vaccines in 2023 [31], representing ongoing advancements in this field (Figure 1).

EVs mainly consist of lipids, proteins, and nucleic acid components [32, 33] (Figure 1). The phospholipid bilayer, which forms the outer layer of EVs, serves the dual purpose of safeguarding the contents and acting as a carrier for drug delivery. The lateral vibration of the phospholipid bilayer is induced by the fatty acid tails of the phospholipid molecules to maintain the dynamic flexibility of the EV structure. Furthermore, cholesterol is present in the lipid bilayer along with surface‐adherent proteins, which enhances the structural rigidity of EVs and their overall stability. The nucleic acid content within EVs encompasses genetic materials, such as DNAs (double‐stranded DNAs, single‐stranded DNAs, mitochondrial DNAs) and RNAs (mRNAs, microRNAs, small nuclear RNAs, non‐coding RNAs, small cytoplasmic RNAs). Transport of these constituents can modulate the expression of genetic information in target cells, thus offering the potential for disease treatment. Lipid components in EVs predominantly include phosphatidylserine, cholesterol, sphingolipids, and ceramides. Variations in the lipid composition of EVs have been used for both disease diagnosis and treatment [34]. EV proteins can either adhere to the surface of the phospholipid bilayer or exist within the internal vesicle cavity. They encompass diverse categories, including signal proteins, enzymes, cytoskeletal proteins, chaperone proteins such as heat shock protein 90 (HSP90) and heat shock protein 70 (HSP 70), and multivesicular bodies (MVBs). Their functionalities are involved in the regulation of EV generation, the mediation of intercellular communication, and the modulation of physiological and pathological processes of the body.

### 2.2 EV Biogenesis

**FIGURE 2:**
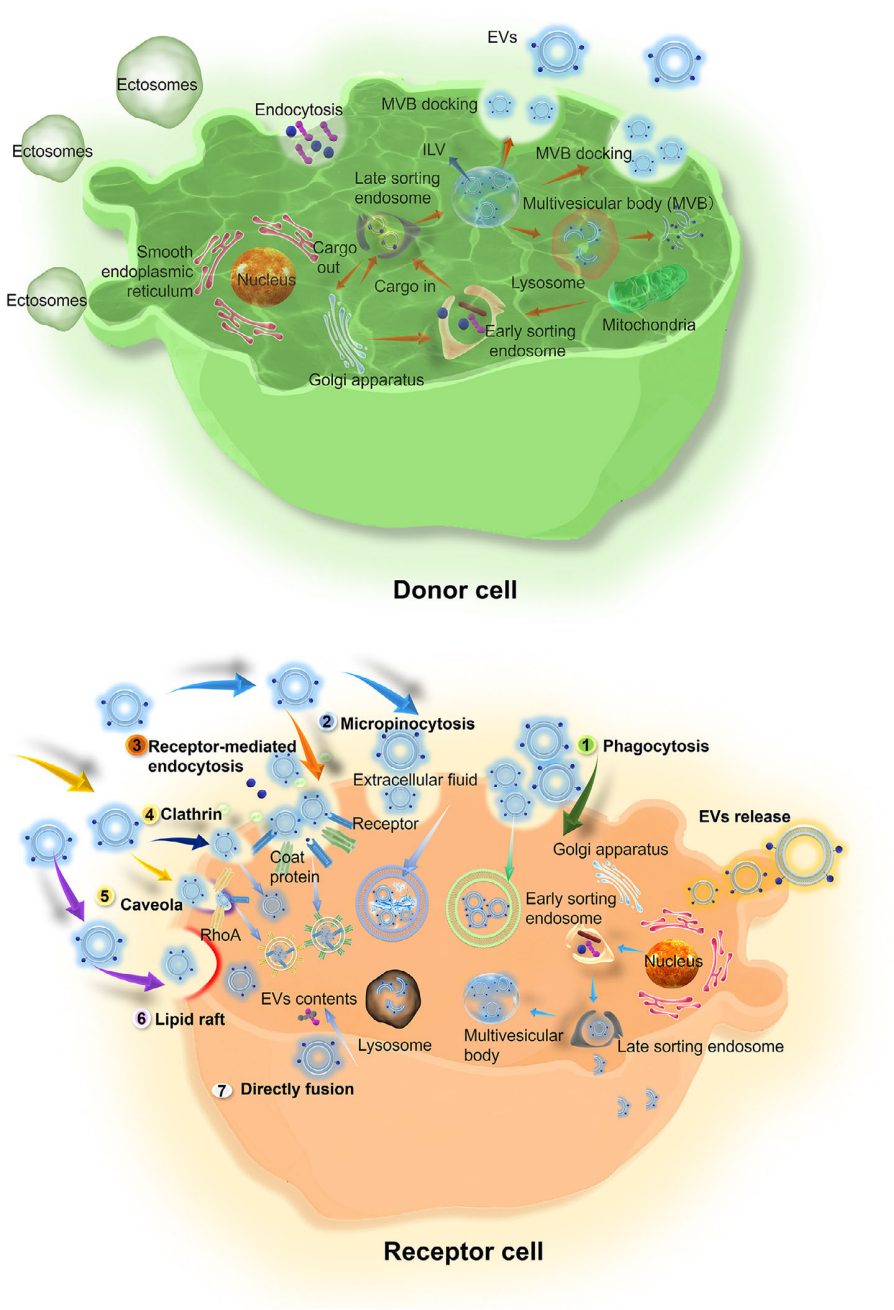
EV biogenesis and uptake. The invagination of the plasma membrane generates EEs, which then mature into LEs and form MVBs under the influence of Rab5, VPS34/p150, endoplasmic reticulum, and trans‐Golgi network. The ESCRT apparatus, comprising ESCRT‐0, I, II, and III, plays an important role in this stage, responsible for recognizing ubiquitinated cargoes, preventing their degradation, and facilitating the formation of ILVs. ESCRT‐0 initiates the sorting mechanism, while the Alix protein delivers non‐ubiquitinated cargoes to ILVs. Additionally, non‐ESCRT mechanisms, such as microstructural domains enriched in nSMase2, promote content sorting through endosomal membrane segregation. nSMase2‐generated ceramides assist in the inward growth of multivesicular bodies (MVBs). As a result, MVBs fused with lysosomes to degradation or fused with the plasma membrane to release EVs. The process of EVs uptake involves multiple mechanisms, including phagocytosis, micropinocytosis, receptor‐mediated endocytosis, clathrin‐mediated endocytosis, caveola‐mediated endocytosis, lipid raft‐mediated endocytosis, and fusion.

EV biogenesis comprises three key stages: (1) the invagination of the plasma membrane generates early endosomes (EEs), (2) EEs mature into late endosomes (LEs) and form multivesicular bodies (MVBs), and (3) MVBs fuse with the plasma membrane to release EVs (Figure 2). The first step is the generation of EEs based on the fusion of primary endocytic vesicles and the invagination of the plasma membrane. EVs are cup‐shaped structures containing extracellular fluid and cell‐surface proteins [36, 37]. Some studies indicated that the endoplasmic reticulum and trans‐Golgi network contributed not only to the biogenesis but also to the composition of the EEs [12, 38, 39]. Subsequently, EEs mature into LEs or are returned to the plasma membrane. Rab5 and its effector VPS34/p150 play a key important role in the conversion of EEs to LEs. In certain instances, intraluminal vesicles (ILVs) are formed within the LEs, resulting in the formation of MVB [40]. The ESCRT apparatus, composed of ESCRT‐0, ESCRT‐I, ESCRT‐II, and ESCRT‐III, plays an important role in the second stage of EV biogenesis. It not only recognizes ubiquitinated cargoes and inhibits their degradation but also induces membrane deformation and protein sorting to generate ILVs. ESCRT‐0/I/II constitutes a strong recognition domain with a high affinity to ubiquitinated cargoes [41]. They sequentially recognize ubiquitin‐modified loaders, with ESCRT‐0 initiating the sorting process and playing a major role [42].

The complex on the endosomal membrane undergoes sprouting, and with the assistance of ESCRT‐III, convergence results in the pinching of the membrane. Subsequently, the buds are released into the endosomes [43]. Alix serves as a pivotal marker protein for EV biogenesis, which facilitates the delivery of non‐ubiquitinated cargoes to the ILVs by interacting with ESCRT‐III. Aside from the ESCRT mechanism, the formation of ILVs is also governed by non‐ESCRT‐dependent pathways. Microstructural domains enriched in neutral sphingomyelinase (nSMase2) facilitate the sorting of EV contents through lateral segregation of endosomal membranes. Ceramides, generated by nSMase2, induce spontaneous negative curvature of the endosome membrane, thereby promoting the internal growth of MVBs.

While MVBs containing ubiquitinated cargoes may be destined to fuse with lysosomes for degradation, there exists an alternative pathway where MVBs fuse with the plasma membrane, leading to the extracellular release of ILVs as EVs. The process is predominantly facilitated by some key regulators, such as Rab27A/B, which guide MVBs to the plasma membrane. This fusion process is orchestrated by the SNARE complex and further activated by the interaction of synaptotagmin with syntaxin. The formation of the trans‐SNARE complex, comprising V‐SNARE on the MVB and T‐SNARE on the plasma membrane, is instrumental in discharging EVs into the extracellular milieu [36, 44]. Notably, some specific cargo was also loaded in Ectosomes, which were produced by the rapid outward curvature and outward budding of the microdomain and then were released to the extracellular fluid [45].

The biogenesis of EVs involves the coordinated action of various organelles and proteins, directly impacting the efficient loading and delivery of mRNA. In the early stages of EV formation, RNA‐binding proteins play crucial roles in the sorting of mRNA into exosomes. YBX1 (Y‐box Binding Protein 1), a multifunctional RNA‐binding protein, can directly bind to RNA molecules, recognizing specific sequences or structures, thereby participating in RNA sorting. YBX1 also interacts with other RNA‐binding proteins to form complexes, which collaboratively facilitate RNA encapsulation [46]. hnRNPA2B1 (Heterogeneous Nuclear Ribonucleoprotein A2/B1) selectively sorts RNA into endosomes by recognizing and binding specific RNA sequences or structures such as EXO‐motifs. Additionally, hnRNPA2B1 binds to polyubiquitin chains and interacts with other RNA‐binding proteins to ensure the selective encapsulation of specific RNA molecules into ILVs [47]. Although these two natural binding proteins do not specifically target mRNA for sorting, they play a significant role in enhancing mRNA loading. Notably, some mRNA‐binding proteins have been shown to facilitate mRNA incorporation into EVs by interacting with exosomal surface proteins. Examples include human antigen R (HUR) and others [48]. Therefore, future research could focus more attention on RNA‐binding proteins to enhance mRNA loading efficiency.

### 2.3 EVs Internalization Pathways in Receptor Cells

The EVs' internalization pathways in receptor cells is a highly complex process but primarily involves endocytosis and directly fusion. Endocytosis includes phagocytosis, micropinocytosis, receptor‐mediated endocytosis (RME), which encompasses pathways such as clathrin‐mediated endocytosis (CME) and caveola‐mediated endocytosis, and lipid raft‐mediated endocytosis. (Figure 2).

Phagocytosis, a mechanism of EV uptake, plays a pivotal role in clearing foreign substances by immune cells. Various cells, including macrophages and dendritic cells (DCs), engage in phagocytosis to engulf EVs [49, 50]. Micropinocytosis is characterized by the uptake of larger quantities of extracellular materials. During this process, the plasma membrane forms an invagination through the driving capacity of actin filaments [51]. Additionally, EV surface protein could bind to the receptor of the cell membrane to mediate the uptake process. When the EV was treated with proteinase, the EV uptake was reduced. EV proteins can modulate circulating kinetics, and the surface composition can be affected by the cellular physiological state. For instance, EVs subjected to protease treatment prior to intravenous administration exhibited delayed clearance. The glycoprotein, a main EV protein, alters the style of recipient cells when the native glycosylation is disrupted [52]. Membrane‐bound receptors significantly influence EV uptake. To be exemplified, the interaction between integrin‐tetraspanin complexes or ephrin receptors (Eph) with cellular receptors enhances EV internalization [53]. Macrophage receptors with collagenous structures (MARCO) also facilitate the uptake of EVs by macrophages [54]. Furthermore, EVs surface protein highly affects the internalization of EVs [55]. For example, EVs surface protein cleaved by low temperature inhibits the EV uptake [56]. Conversely, proteins like ICAM‐1 and CD9 have been shown to promote internalization through interactions with specific receptors on target cells [57]. The preferential uptake of smaller EVs and the crucial role of specific surface modifications in targeting recipient cells underscore the complexity of EV internalization. For instance, modification of rabies viral glycoprotein on EVs enables crossing the BBB and targeting the brain [30, 58], while α6β4 and α6β1 integrins on EVs facilitate uptake by lung‐resident fibroblasts. Similarly, EVs with αvβ5 integrins related to liver metastasis are preferentially taken up by Kupffer cells, which enhances their targeting to the liver [59].

CME, as a specific subtype of RME, is a prevalent pathway for EV uptake, where inhibition of clathrin expression significantly diminishes EV internalization [49]. Beyond clathrin, proteins such as caveolin1, RhoA, and ARF6 are pivotal in creating specific microdomains (caveolae or lipid rafts) that facilitate EV entry [19]. Fusion represents another pathway for EV internalization that involves the close approach of two lipid bilayer membranes. This results in the formation of a hemifusion stalk that expands to create a bilayer hemifusion diaphragm. This process culminates in creating a fusion pore, merging their hydrophobic cores and allowing EV entry into recipient cells [49].

### 2.4 EVs as Delivery Vectors

EVs have emerged as potential delivery vehicles due to their unique biological properties, such as biocompatibility and low immunogenicity, and so forth. Depending on these advantages, EVs that encapsulate a wide range of therapeutic molecules, such as chemical drugs, proteins, and nucleic acids are ideal candidates for therapeutic applications.

For chemical drugs that have poor stability and significant side effects, EVs can enhance their stability, targeting ability, and reduce the immunogenicity of these drugs. For example, paclitaxel (PTX), a chemotherapeutic drug with poor water solubility, was loaded into milk‐derived EVs by Agrawal et al., extending its release time to 48 h and significantly improving its water solubility and stability. Notably, EV‐loaded PTX reduced systemic inflammatory response, cytokine storm compared to intravenous PTX administration [60]. EVs are also effective carriers for protein and peptide molecules, particularly in cancer immunotherapy. For instance, EVs derived from DCs loaded with melanoma antigen exhibited promising clinical outcomes in treating non‐small cell lung cancer [61]. The delivery of nucleic acid drugs via EVs is a rapidly developing field. Nucleic acid drugs showed superior therapeutic potential in many diseases, despite their drawbacks such as not crossing biological barriers, a lack of targeting capability, and being unstable in the in vivo environment. EVs, as natural endogenous nanocarriers, can address these issues, especially for mRNA drugs. For example, RBD (receptor‐binding domain) mRNA encapsulated in milk‐derived EVs constituted a new oral vaccine for SARS‐CoV‐2, resulting in the successful induction of neutralizing antibodies in mice [23].

EVs are particularly promising delivery vesicles for mRNA delivery due to their natural ability to encapsulate mRNA and protect it from degradation, coupled with their efficiency in cellular uptake and cytoplasm release. Preclinical studies have demonstrated the significant therapeutic benefits of EV‐mediated mRNA delivery in treating cancers, genetic disorders, and infectious diseases [62]. Leveraging their unique properties, EVs can effectively transport mRNA to target cells, ensuring their stability and maintaining their functionality. This approach enhances the stability, bioavailability, and efficacy of mRNA, highlighting the potential of EVs as a superior mRNA delivery platform.

## 3 Advantages of EV‐Based mRNA Therapeutics and Vaccines

**FIGURE 3:**
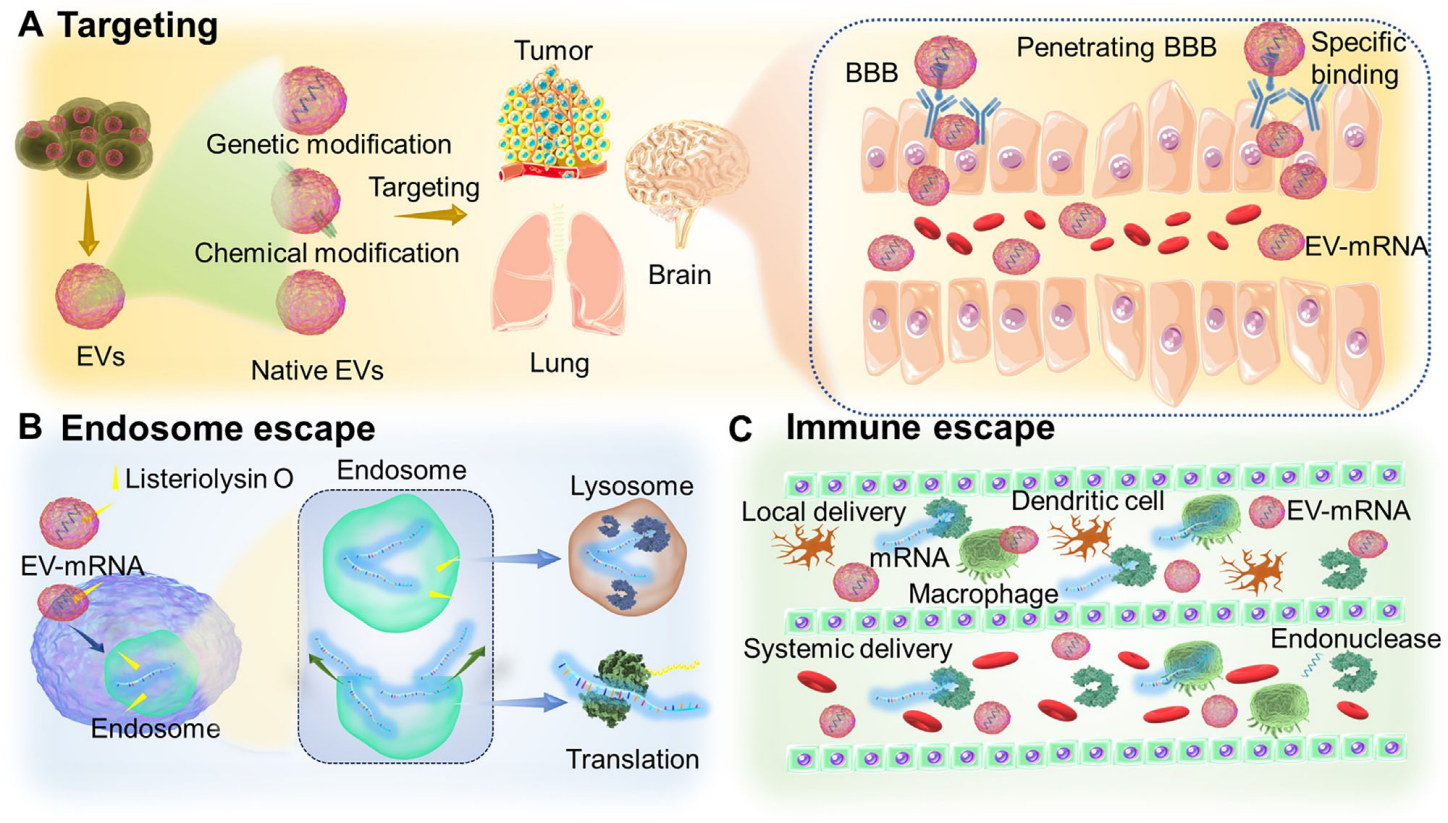
Advantages of EV‐based mRNA therapeutics and vaccines. (A) EVs derived from various cells have natural targeting‐like tumor homing effect. Genetic and non‐genetical modification further strengthen the targeting, resulting in EVs targeting tumors, the brain, and the lung. EVs are modified with peptides, including RVG, iRGD, and c(RGDyK), to penetrate BBB. (B) Listeriolysin O, a special protein with the ability to accelerate endosomal membranes to form pores, is connected to EVs to enhance the translation efficiency of mRNA and endosome escape. (C) Owing to the protection of EVs, mRNAs can avoid the clearance of macrophages and endonucleases within the body.

Efficient in vivo mRNA delivery, sufficient translation efficiency, and avoidance of unwanted immune responses are critical requirements for optimal performance of mRNA therapeutics and vaccines. However, intracellular delivery of exogenous mRNA encounters significant obstacles, such as susceptibility to enzymatic degradation and challenges posed by the lipid membrane barrier due to electrostatic repulsion. Moreover, achieving targeted mRNA delivery to specific organs, tumors, or cells remains a pressing technical challenge. EVs, as naturally occurring vesicles, offer a promising solution to these challenges by encapsulating mRNAs. Precision engineering modification of EVs enables the specific targeting of mRNA to distinct tissues and organs, a great advantage in overcoming challenges inherent to conventional delivery systems. The capability to cross barriers like the BBB, tumor barrier, and membrane barrier signifies a transformative stride toward mRNA delivery and offers novel prospects for therapeutic applications. Diminished immunogenicity ensures the preservation of encapsulated mRNA integrity, which minimizes systemic toxicity often associated with nucleic acid‐based drugs. Sufficient mRNA translation and endosome escape achieved by engineered EVs ensure the therapeutic efficacy of mRNA (Figure 3).

The widespread clinical application of mRNAs largely hinges on precise targeted delivery, which can amplify their local concentrations while minimizing side effects. Natural EVs exhibit an excellent homing effect with preferential affinity toward parental cells. Intercellular adhesion molecule‐1 (ICAM‐1) is a common protein on the surface of EVs, which can intact with its receptor on the acceptor cells, thus facilitating cellular uptake or crossing through biological membrane barriers (i.e., BBB) [18]. The natural targeting ability of EVs is generally insufficient. Therefore, it is necessary to fabricate engineered EVs to enhance their targeting properties through surface modifications involving either genetic or non‐genetic approaches. As for genetic engineering, the gene sequence of the targeting protein or peptide was commonly fused to the membrane protein sequence of the donor cell, and the EVs with the targeting protein were subsequently secreted [63]. For instance, RVG peptide fused to Lamp2b, an EV anchor protein, enables specific targeting to neurons and microglia [30], and iRGD peptides fused to Lamp2b on EVs improve their targeting to cancer cells [64]. Although genetic engineering is effective and specific for the expression of proteins or peptides, it is limited by the fewer targeting ligands. Non‐genetical engineering, such as chemical modification through techniques like click chemistry, enables the attachment of additional ligands to EV surfaces, thus potentially improving their targeting ability. For example, the c(RGDyK) peptide was used to modify EVs using click chemistry. RGDyK‐EVs showed highly efficient penetration for BBB. In another study, EVs modified with neuropilin‐1‐targeted peptide by cycloaddition reaction could also penetrate BBB and obtain the ability to target tumors [65, 66]. In contrast to genetic engineering, non‐genetical engineering relies on bioconjugation and lipid insertion and allows for more ligands to be anchored on the surface of EVs. However, the site specificity and the efficiency of the conjugation reactions may be lower than in genetic engineering [63]. The biosafety and stability of EVs may also increase due to the addition of chemical reagents. Despite these challenges, targeted EV delivery has been extensively explored to date.

Most mRNAs are degraded by the immune system, and only 0.01% of them can enter target cells [67, 68]. Due to their highly charged nature and large size, it is difficult for mRNA molecules to traverse the plasma membrane. LNPs encapsulating ionizable lipids are internalized by cells owing to their inherent positive charge, which facilitates fusion with the negatively charged cell membrane. Once inside the cell, a reduction in pH triggers a structural alteration in the ionizable lipids, resulting in the disintegration of LNPs and subsequent mRNA release. Currently, LNPs are recognized as a primary delivery vector for mRNAs. However, the potential toxicity related to the lipid components and repeated administration in the short term is an unavoidable problem for LNPs. In contrast, EVs as endogenous vesicles exhibit lower immunogenicity, reduced toxicity, natural targeting, and enhanced biocompatibility compared to LNPs, thus supporting their use in repeated mRNA dosing for clinical trials [67]. For example, EVs provided sustained mRNA delivery over multiple injections without attenuation of the expression signal or adverse injection site responses, indicating better stability and durability as a delivery vehicle. Meanwhile, EVs demonstrated a higher encapsulation efficiency of mRNA (approximately 90%) compared to LNPs. This higher efficiency is crucial for effective mRNA delivery [69]. Besides, EVs derived from MSCs are well‐tolerated and have few side effects when repeatedly injected. These indicated that EVs have promising therapeutic applications by themselves [70]. Compared with the LNP‐based mRNA vaccines, EV‐encapsulated mRNA vaccines elicited stronger immunoglobulin and lgA responses [35], as EVs could protect mRNAs from the degradation of blood‐derived ribonucleases while CD47, the membrane protein on the surface of EVs, releases a “don't eat me” signal, avoiding the phagocytosis of macrophage cells [71, 72]. Another study indicated lung‐derived EVs have better cellular targeting and molecular function, greater lung distribution and retention, higher mRNA translation, and protein expression in the target organs than LNP‐based mRNA [73]. Notably, most liposomes achieve endosomal escape in tumor cells by disrupting the stability of the endosomal compartments, which results in the effective release of their cargo primarily in the outer layers of tumor tissue. Recent studies, however, have shown that EVs can remain stable within the endosome and release their cargo upon interacting with the endosomal membrane. This suggests that EVs are biocompatible and can naturally fuse with cellular membranes, facilitating more efficient mRNA delivery [74].

Apart from LNPs, viral vectors, polymer‐based nanoparticles, and inorganic nanoparticles can also be used for mRNA delivery. Adeno‐associated viruses (AAVs), due to their high infection efficiency, are effective but may trigger unwanted immune responses [75], and their toxicity and size limitations restrict further development [76]. Inorganic nanoparticles, such as dendrimer‐modified gold nanoparticles, are used for intracellular mRNA delivery, protecting mRNA from nuclease degradation [77]. Polyethyleneimine (PEI)‐modified graphene quantum dots are also utilized for mRNA delivery [78]. Despite their good stability and ease of functionalization, inorganic nanoparticles still pose potential toxicity issues due to their undegradable property. Polymer‐based nanoparticles often use cationic polymers to compact negatively charged mRNA into nanoparticles. Commonly used cationic polymers include PEI [79], polylysine [80], and dendrimers [77]. For instance, PEI‐modified oxidized graphene successfully delivered mRNA encoding reprogramming transcription factors to pluripotent stem cells, significantly improving the reprogramming efficiency of human induced pluripotent stem cells, and successfully generated rat and human induced pluripotent stem cells from adult adipose tissue‐derived fibroblasts [81]. However, the large molecular weight of cationic polymers makes them difficult to biodegrade, and their high number of amine groups results in a strong positive charge, which can often lead to additional cytotoxicity. Thus, the above vectors still present toxicity and safety concerns. However, EVs, as endogenous nanovesicles, offer superior safety profiles. Moreover, EVs possess some unique advantages, such as targeted homing abilities and the innate capacity to cross physiological barriers, which are not present in other delivery systems.

Additionally, sufficient translation and pharmaceutical performance of mRNAs are hampered because most mRNAs may be degraded by endosome‐lysosome compartments after entering target cells, leading to little mRNA translation into functional protein to exert a therapeutic effect. Listeriolysin O, a special protein with the ability to accelerate endosomal membranes to form pores, is connected to EVs containing functional mRNA. The mRNA, assisted by Listeriolysin O, circumvents endosomal degradation, thereby significantly enhancing translation efficiency [82].

## 4 Challenges of EV‐Based mRNA Therapeutics and Vaccines

Despite the promising potential of EV‐based mRNA therapeutics and vaccines, several significant challenges remain that must be addressed to ensure their successful clinical application. These challenges include issues related to scalable production, efficient mRNA loading, storage, and stability, and more specific targeting of EV‐based mRNA. Addressing these obstacles is crucial for advancing the development and clinical translation of EV‐based mRNA therapies and vaccines.

Scalable production of EVs is challenging for EV‐based mRNA therapeutics and vaccines, and limited EV production hampers its clinical transformation. Current production methods mainly involve the isolation and purification of EVs from cell culture supernatants, which is costly and unsuitable for large‐scale manufacturing. However, several approaches are laying the foundation for the large‐scale production of EVs. Cellular nanoporation could stimulate cells to obtain large‐scale EVs. Notably, not only were EVs increased, but also more mRNA was encapsulated by EVs [4]. Additionally, three‐dimensional dynamic culture and bioreactors also increase the production of EVs. Because there is more area for cells to grow, large‐generation EVs are obtained under these two methods [83, 84]. The efficient secretion of EVs can also be promoted by interfering with the related proteins in the secretion process of EVs. For example, aptamer‐like protein KIBRA inhibited the ubiquitination degradation of Rab27a and promoted EVs secretion [85]. Challenges such as material loss during purification, standardization of production conditions, and cost‐effectiveness analysis are limiting further development of large‐scale EV production. Therefore, optimizing and addressing these issues should strengthen future EV research.

### 4.1 Efficient mRNA Loading

In the exogenous loading approach, electroporation enhances the delivery efficiency of cargo molecules like small‐molecule drugs and small interfering RNAs (siRNAs) into EVs. However, due to the large molecular weight of mRNA, electroporation may not be the optimal choice, as the size and stability of mRNA hinder their efficient loading into EVs [86]. Endogenous loading approaches involve a transfection process typically utilizing viral vectors or plasmids. Although this approach can enhance the efficiency of endogenous mRNA loading, it tends to be relatively inefficient, with a loading rate of approximately 1‰ [87]. To improve mRNA loading efficiency in EVs, researchers are exploring the utilization of sorting proteins (RNA‐binding proteins), such as MS2P, HUR and L7Ae [48, 88, 89]. These sorting proteins can selectively package mRNAs into EVs during their biosynthesis by recognizing and binding to specific RNA sequences or structures. This innovative approach holds promise for enhancing mRNA loading efficiency in EVs, thereby expanding their potential for diagnostic and therapeutic applications.

### 4.2 Storage and Stability

Maintaining the stability of EVs and encapsulated mRNA during storage and transportation is also a challenge for EV‐based mRNA therapeutics and vaccine clinical translation. EVs are best stored at ‐80°C to preserve their biological activity [90]. However, the practical limitations of deeply freezing storage in real‐world settings pose a significant challenge to the advancement of EV‐based mRNA therapies. To address the issue of storage stability, researchers have explored freeze‐drying technology to prepare EV‐based mRNA as a dry powder system. This approach has notably extended the shelf life of EV‐based mRNA to 28 days, offering a potential solution to the storage and stability concerns associated with EV‐based therapeutics [21].

### 4.3 Specific Targeting

Although EVs have inherent targeting capability, achieving targeted delivery of EV‐based mRNA to specific cells or tissues remains a challenge for EV‐based mRNA therapeutics and vaccines. Through cellular engineering aimed at bolstering the expression of anti‐HER2 antibodies within EV‐based mRNA, researchers have attained heightened targeting efficacy against breast cancer with abundant HER2, thereby curbing cancer progression [28]. Drawing from this precedent, the surface of EVs can be tailored to exhibit highly expressed antibodies corresponding to disease‐associated proteins, enabling precise targeting of EV‐based mRNA therapies. Moreover, the incorporation of anti‐CD71 and PD‐L1 antibodies into EV‐based IFN‐γ mRNA, as well as the modification of EV‐based TP53 mRNA with tissue‐targeting peptides, has bolstered the targeting prowess of EV‐based therapeutic systems [48, 91]. Hence, the ongoing advancement of novel targeting methodologies represents a pivotal avenue for achieving precise targeting of EV‐based mRNA therapeutics and vaccines.

## 5 Strategies for Loading mRNAS in EVs

**FIGURE 4:**
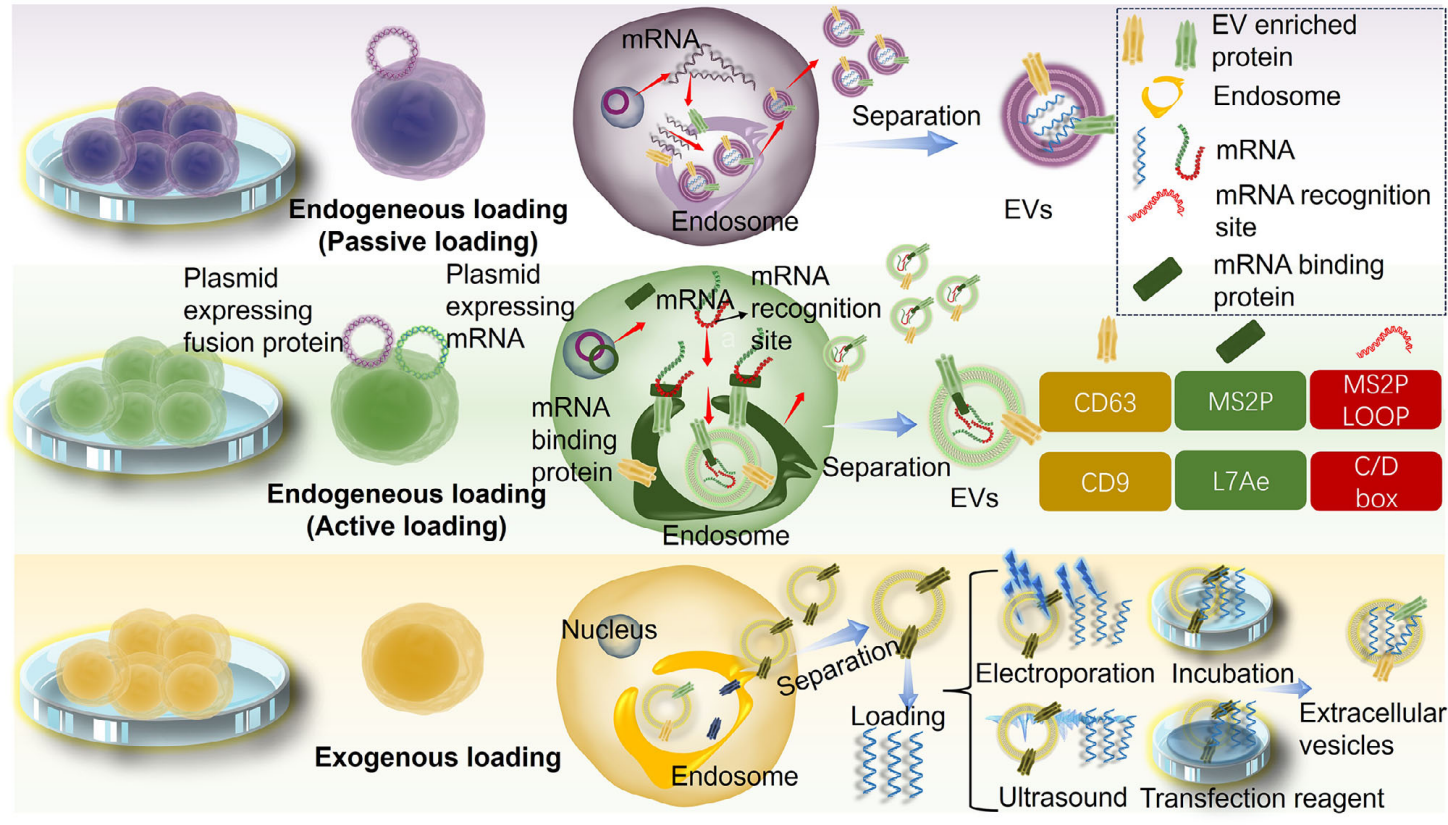
Packaging strategies for loading mRNAs in EVs. mRNA can be loaded to EVs by endogenous mRNA loading (passive loading and active loading) and exogenous mRNA loading.

Owing to their natural penetration of physiological barriers, low immunogenicity, and biocompatibility, EVs have gained more attention in mRNA delivery. Meanwhile, the COVID‐19 pandemic further enhanced the potential of the mRNA vaccines [92]. Significantly, both the strong membrane‐binding capacity and better cytoplasticity of EVs facilitate the interaction of mRNA with EVs [87]. Here, packaging strategies and regulatory factors of mRNA in EVs are introduced. Packaging strategies for mRNA loading into EVs can be divided into endogenous and exogenous mRNA loading [89]. Endogenous mRNA loading refers to the incorporation of mRNA into EVs during EV biogenesis, and exogenous mRNA loading relies on electroporation, ultrasound, incubation, and chemical transfection reagents to package mRNAs into EVs (Figure 4).

Based on how mRNAs are loaded into EVs, endogenous mRNA loading is divided into passive and active loading. Plasmid transfection is the most common way for passive mRNA loading. Plasmid, containing the target RNA sequence, is introduced into the specific cells, and mRNAs are subsequently released. As a result, mRNAs are translated into therapeutic proteins or induce repression of certain genes or viruses. For example, macrophage cells were transfected by IRES‐IL‐10 plasmids to produce IL‐10 mRNA which controlled atherosclerosis treatment. IL‐10 mRNA was not only efficiently delivered into inflammatory cells for specific translation induction but also alleviated the local inflammation and had therapeutic effects in ApoE mice [93]. In another study, tumor cells were transfected with the plasmids encoding the suicide gene, constructed to produce the EVs with suicide mRNA. As a result, these EVs induced tumor cell apoptosis and inhibited the growth of tumors when applied to them [94]. Although the loading of mRNAs is successful with the assistance of a plasmid, the instability and short expression period hamper its further application. Yang et al. stimulated cells to obtain a large generation of EVs encapsulating mRNAs by the cellular nanoporation biochip. A larger number of pores were produced in the plasma membrane under the electric field, and these pores closed after 2 min. Large amounts of DNA were injected into donor cells, and MVBs were thus significantly added during this process. Compared with conventional loading methods like electroporation, the mRNA loading efficiency significantly increased about 1000‐fold in EVs, while a 50‐fold increase in EV production was achieved [4]. Owing to the simplicity of operation, passive loading is the most common way of packaging mRNAs in EVs. However, the limitation to specific cell types of plasmid transfection remains the major issue for passive loading.

**FIGURE 5:**
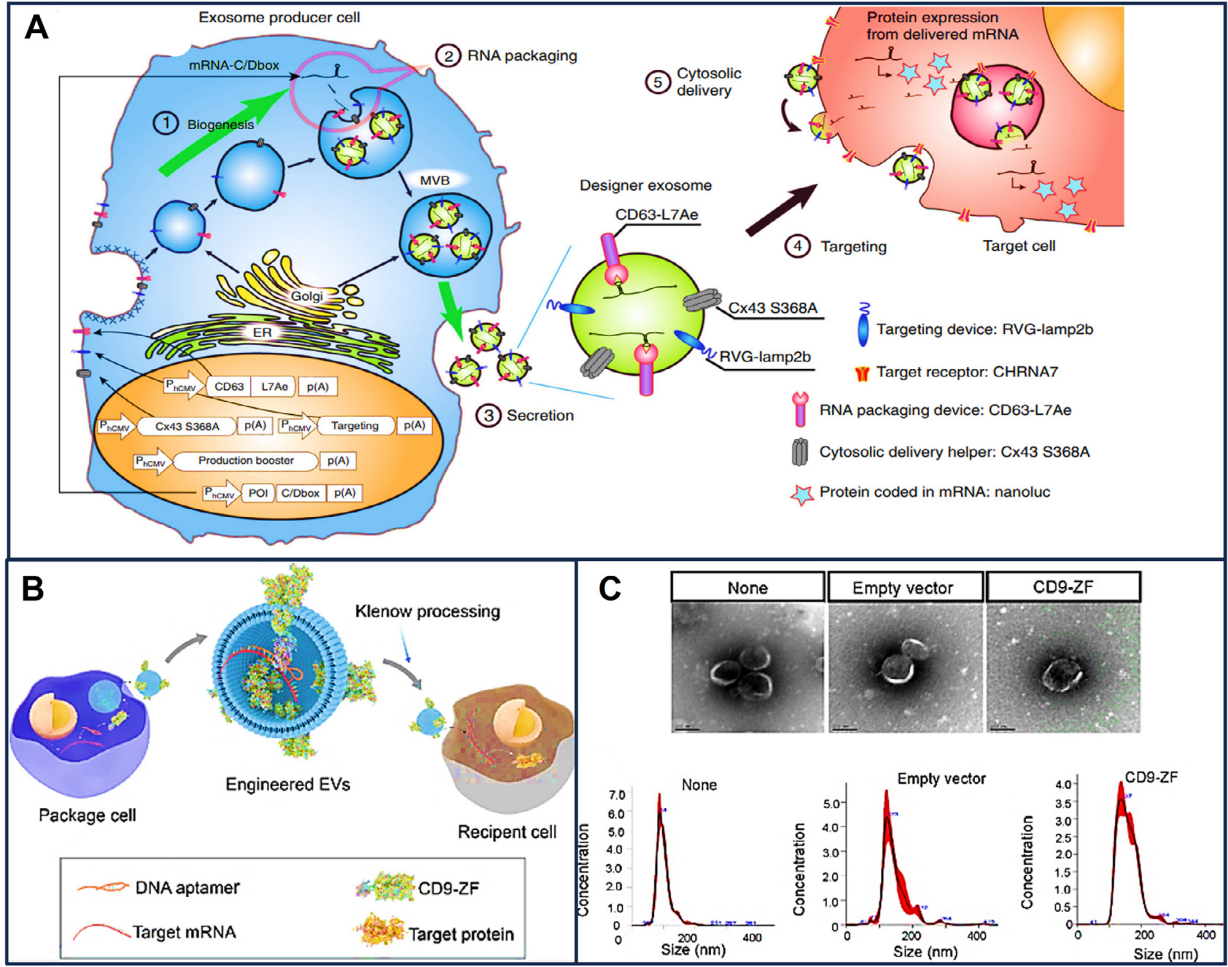
Endogenous mRNA actively loaded into EVs. (A) Fusion protein and (B) DNA aptamer are used to combine mRNAs with EVs. Copyright 2018, Springer Nature. (C) Morphology and particle size of EVs loading functional mRNA. Reproduced with permission [88, 96]. Copyright 2021, American Chemical Society.

Active loading, as a more efficient method to load mRNAs into EVs, is developed in the context of the limitation to specific mRNA packaging for passive loading. Active loading is generally achieved by transfecting two types of plasmids, thereby achieving effective mRNA loading. The fusion proteins, as products of one type of plasmid transfection, could bind to EV membrane proteins like CD9 and CD63. Meanwhile, the fusion protein could also be intact with the specific RNA structure. Especially, one type of fusion protein could bind to specific RNA depending on the corresponding RNA elements [95]. Owing to the interaction of fusion protein to EVs and mRNAs, large amounts of mRNAs of interest were specifically loaded into EVs. For example, mRNAs were then overexpressed in EVs from donor cells. L7Ae, the archaeal ribosomal protein that can bind to the RNA structure, was conjugated to the CD63 protein on the surface of EVs. As a result, mRNA was 100‐fold higher than in the normal transfection. Meanwhile, other cargos‐like proteins also increased in EVs because of the cytosolic delivery helper [88] (Figure 5A). In another experiment, the fusion protein HuR was fused with the EV membrane protein CD9 and bound to the RNA with high affinity. Therefore, CD9‐HuR EVs were efficiently delivered to the receptor cells and successfully recognized the targets. Notably, combining the N peptide and the box B sequence is also a method to actively obtain mRNAs. EVs, attached with CD64 protein fused with N peptide, actively linked IFN‐γ mRNA with box B sequence, resulting in efficient translation of mRNA [48]. Besides the fusion protein, DNA aptamer is also used to combine mRNAs with EVs. Unlike fusion protein, DNA aptamer not only could specifically bind to the relevant region of the start codon AUG of interest mRNA but also could be recognized by zinc finger motifs fused with the CD9 on the surface of EVs [96]. Corresponding studies demonstrated that the PGC1 α mRNA with a 2.5‐fold increase was loaded into EVs [96] (Figure 5B,C). In conclusion, fusion proteins like L7Ae, HuR, and N peptide or DNA aptamer were designed as a bridge to connect interest mRNA with EVs. Therefore, more mRNAs were specifically sorted into EVs. Despite the loading efficiency of mRNA of interest in EVs being significantly increased, the sustainable mRNA expression and the multiple categories of cell transfection remain unsolved issues.

Electroporation is a widely used method to load mRNAs into EVs in exogenous loading. Temporary pores are created in the cell membrane and allow interest mRNA to enter the target cells. The diameter of pores or dimples on the surface of the plasma membrane ranges from 2 nm to a maximum of several nanometers. Notably, large amounts of hydrophobic pores are transformed into hydrophilic pores because the energy is reduced with gradually increasing membrane voltage. Hydrophobic pores need more energy to maintain than hydrophilic pores [97]. For example, SARS‐CoV‐2 Spike (S) protein mRNA was loaded into the lung‐derived EVs by electroporation. Compared with S‐liposomes, S‐EVs elicited stronger immunoglobulin and secretory lgA reactions. In addition, S‐EVs also have a stronger distribution to bronchioles because of the natural lung tendency [21]. FOXF1 mRNA was also loaded into EVs with the assistance of electroporation for treating intervertebral disc (IVD) degeneration. EVs loading FOXF1 mRNA, as the invasive treatment for intervertebral, can significantly increase the healthy factors like keratin 19 and downregulate the IL‐1β in IVD mice cells [98]. Although electroporation is sustainable, the aggregation of mRNA or proteins in EVs cannot be ignored.

In addition to electroporation, lots of loading methods have been reported for loading mRNAs into EVs, such as incubation and sonication. To be exemplified, Tsai et al. harnessed cell‐derived EVs to load Antares2 mRNA with an encapsulation efficiency of up to 90%. It may be due to the preincubation of mRNA with a solution of cationic lipids that realizes the higher encapsulation efficiency. Besides, the resultant mRNA‐loading EVs remained consistent in size, with the polydispersity under 0.2. As a result, stable and long‐term cellular and humoral immune response was successfully induced by EVs loaded with Antares2 mRNA [99]. As for the vaccine, RBD mRNA was mixed with DOTAP cationic lipids and loaded into milk EVs via incubation and mixing. As a result, an oral vaccine was obtained via the attractive force of water. RBD peptide was produced in cells and thus elicited humoral immunity against SARS‐CoV‐2 in mice [23]. Another study mixed EVs or LNPs with purified two or more mRNAs preincubating with polycationic lipids. Both in cells and live animals, EV‐mRNA has lower adverse effects and promotes longer cellular and humoral immunity than LNP‐mRNA. This study constructed a multiplexed EV‐mRNA vaccine with higher protein expression, greater safety both in vivo and in vitro, and more immunity to multiple antigens than LNP‐mRNA [25].

Ultrasound is also a popular strategy to encapsulate drugs in EVs. Ultrasound can change the integrity of the plasma membrane with mechanical forces, after which it will return to its original state within an hour. Drugs could enter EVs through the temporary membrane opening. Despite ultrasound being mostly applied in chemical medicine like paclitaxel [100], it acts as a physical technology to assist mRNA‐EVs for specific tissue tropism. EVs and microbubbles were simultaneously delivered into mice, and the microbubbles were subsequently destroyed by ultrasound to target specific tissue. With the crushing influence, the cell membrane permeability increases, and more EVs are delivered into recipient cells. Therefore, mRNAs could be activated effectively and minimize the off‐target effects [101]. In addition, focused ultrasound could open the BBB with a noninvasive strategy [102]. Consequently, ultrasound can also be applied for brain‐targeted EV‐loading mRNA delivery.

In addition to the aforementioned loading methods, another approach involves fusion with other particles. This technique for encapsulating mRNA into EVs combines them with other delivery systems, such as liposomes or LNPs, to create hybrid vesicles. The goal is to enhance stability, loading efficiency, and targeting specificity. Fusing EVs with LNPs can significantly improve mRNA stability, preventing premature degradation in vivo. Furthermore, the resulting hybrid vesicles not only increase mRNA loading efficiency but also leverage the targeting modifications of LNPs to achieve precise delivery to specific cell types. Relevant studies have demonstrated the efficient in vivo delivery of ALKBH5 mRNA to colorectal cancer cells using folic acid‐modified EVs‐liposome hybrid nanoparticles (FA‐ELNPs), significantly inhibiting the progression of colorectal cancer [103]. However, this technique faces challenges such as increased complexity in the production process, difficulties in purification, potential toxicity risks, and batch‐to‐batch consistency issues, necessitating further research and optimization.

In summary, packaging mRNA into EVs can be achieved through endogenous and exogenous methods. Endogenous loading includes passive and active loading. Passive loading typically involves plasmid transfection, which is simple, cost‐effective, and suitable for large‐scale EV production. However, it is limited by cell type specificity, with varying transfection efficiencies across different cell types, and may not ensure mRNA stability. Additionally, it may cause off‐target effects, leading to non‐specific expression. Active loading employs fusion proteins or aptamers to promote the sorting of mRNA into EVs, significantly enhancing RNA loading efficiency and allowing for the regulation of different RNA elements, thus increasing loading flexibility. Despite these advantages, the process is more complex, costly, and presents challenges in maintaining mRNA expression. Exogenous methods, such as electroporation, incubation, and sonication, offer different benefits. Electroporation creates temporary pores in the EVs' membrane, enabling mRNA to enter EVs, but may also cause mRNA aggregation and potential damage to EVs. Incubation and sonication are simple methods that can maintain the relative integrity of the EV membrane, though they may result in lower loading efficiency. The advantages of fusion with other particles include enhanced mRNA stability, improved delivery efficiency, and precise targeted delivery. However, this technology faces challenges such as increased production complexity, difficulties in purification, potential toxicity risks, and consistency issues between batches, which need further resolution to promote its clinical applications [4, 104, 105].

## 6 EV‐Based mRNA Therapeutics

**FIGURE 6:**
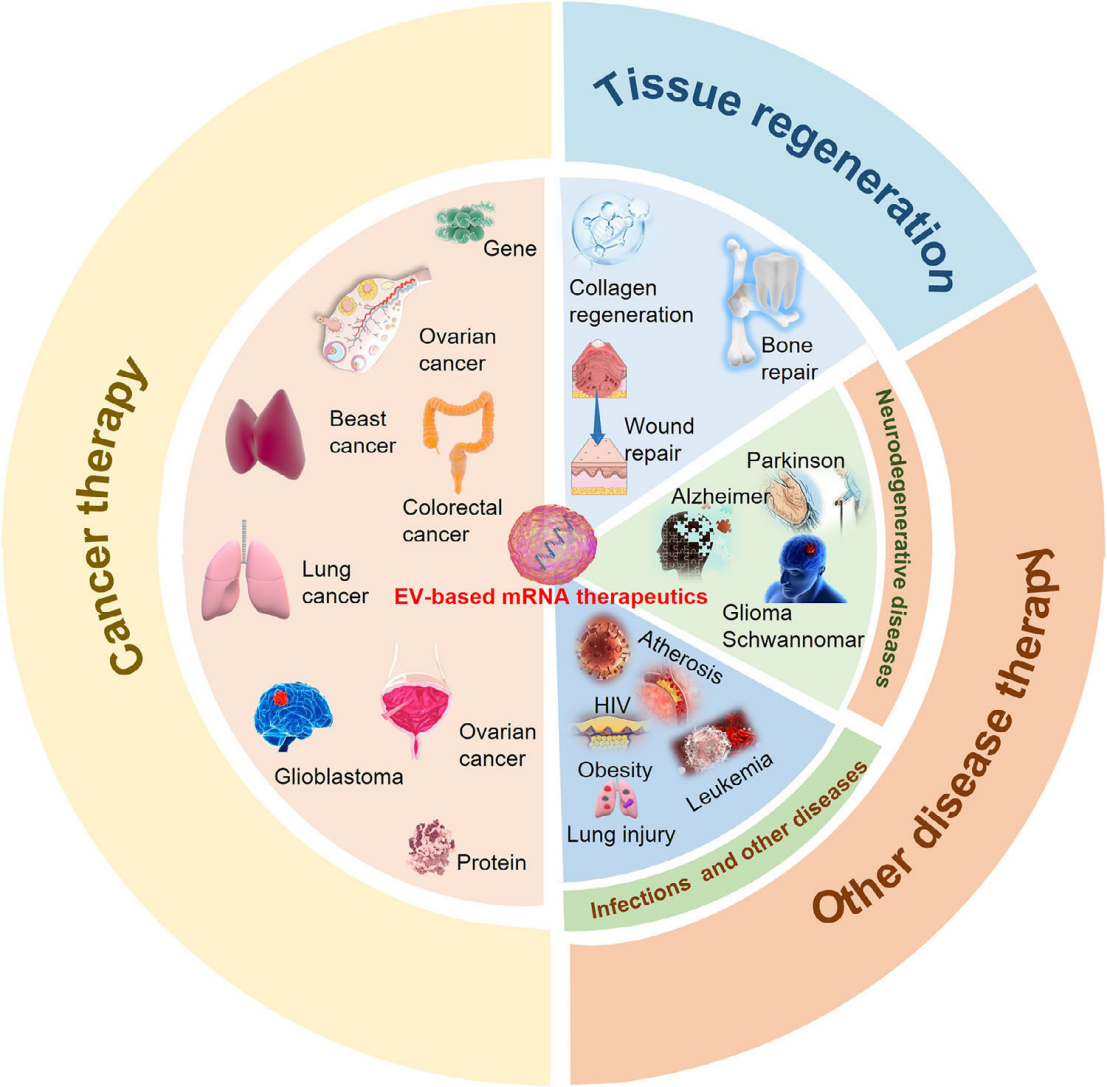
EV‐based mRNA therapeutics.

mRNA is a crucial biomolecule that mediates the transmission of genetic information and governs the process of protein synthesis [106]. Unlike DNA‐based therapy, mRNA‐based therapy offers rapid and efficient protein expression because it directly fulfills its functions in the cytoplasm instead of the nucleus. It is the translation in the cytosol that results in effective gene expression and causes little genome integration and insertion mutation. Furthermore, an interest protein can also be translated by mRNA in tumor‐dormant cells [107, 108, 109, 110]. Owing to the above advantages, mRNA‐based therapy has been applied in various fields such as cancer, regeneration, infectious diseases, and vaccines [111, 112, 113]. However, inefficient delivery of mRNA caused by large volume, instability, and low targeting hampered its precise role in therapy [114, 115]. EVs, as endogenous membrane vesicles and natural delivery carriers, offer promising potential for efficient delivery of mRNA because of their low immunogenicity, specific targeting, and ability to penetrate physiological barriers [39]. mRNA‐based therapy can be mainly categorized into several sections: cancer, tissue injuries, neurodegenerative diseases, infections, hematological, vascular, and obesity diseases (Figure 6).

### 6.1 EV‐Based mRNA for Cancer Immunotherapy

EV‐based mRNA therapy for cancer immunotherapy is currently applied across diverse cancer types. Engineered EVs enhance the specificity, yield, and efficiency of transfection and encapsulation in natural mRNA‐based cancer therapy. Notably, EVs loaded with mRNAs expressing editing proteins provide a platform for gene editing. Additionally, the utilization of EVs carrying mRNAs offers a non‐invasive means of obtaining information about an organism's status. This underscores its potential as a biomarker for cancer and holds promise for rigorous clinical applications [33, 113, 116, 117, 118] (Table 1).

**TABLE 1:** EV‐based mRNA delivery for treatment of cancers.

mRNAs	Administration	Diseases	Therapeutic outcomes	Ref.
HChrR6‐mRNA	Intravenous injection	Breast cancer	Inhibited activity of these cancer cells	[28]
HChrR6‐mRNA	Intravenous injection	Breast cancer	Converted prodrug CNOB into MCHB	[119]
LOCR mRNA	Intravenous injection	Breast cancer	Longer survival and little lung metastasis in model mice	[120]
MMP1 mRNA	Intraperitoneal injection	Ovarian cancer	Facilitating peritoneal dissemination	[121]
LAT1, ASCT2 mRNA	Not described	Colorectal cancer	Promoted cell migration and proliferation	[122]
IFM‐γ mRNA	Intravenous injection	Glioblastoma tumor	Upregulating MHC‐1 to improve cancer immunotherapy	[125]
PTEN mRNA	Intravenous injection	Glioma	Prolonged survival and inhibited tumor growth	[4]
ALKBH5 mRNA	Intravenous injection	Preclinical tumor	Inhibited tumorigenesis and prolonged the survival of Preclinical tumor models by restricting glycolysis	[127]
CD‐UPRT‐EGFP mRNA	Intratumorally administration	Glioblastoma	Triggering of cell apoptosis	[94]
HLA‐DRB1, HAVCR1 mRNA et al.	Not involved	Breast cancer	Biomarker to predict breast cancer	[159]
OATP1B3 mRNA	Not involved	Colorectal cancer	Biomarker to predict colorectal cancer	[160]
VEGF/CD133 mRNA	Not involved	Colorectal cancer	Biomarker to predict colorectal cancer	[161]
SLC2A1/GPRC5A/KRT17 mRNA	Not involved	Bladder cancer	Biomarkers with bladder cancer	[162]
AnxA2 mRNA	Not involved	Bladder cancer	Biomarker for responsiveness to chemotherapy in bladder cancer	[163]
NSD1 and FBXO7 mRNA	Not involved	Gastric cancer	Biomarkers for gastric cancer	[125]

**FIGURE 7:**
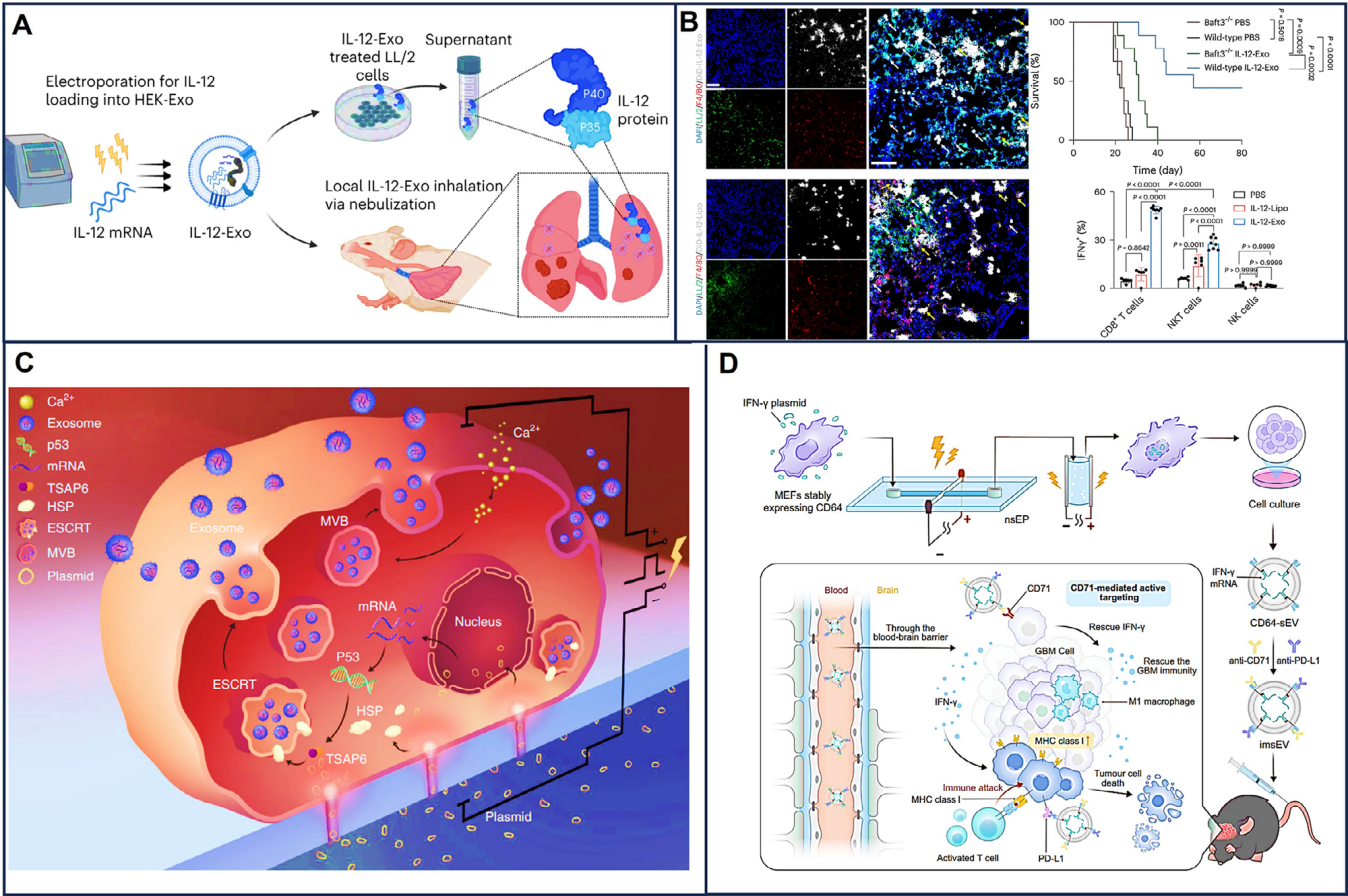
(A) IL‐12‐Exo promotes systemic immunity and was used for the treatment of lung cancer. (B) IL‐12‐Exo has superior lung distribution, promotes the survival of mice and IFNγ expressions on CD8 T cells, NKT cells, and NK cells in the TME (tumor microenvironment) [123]. Copyright 2023, American Chemical Society. (C,D) Cellular nanoporation and the combination of nano‐and milli‐second pulses. Reproduced with permission [4, 48]. Copyright 2018, Springer Nature; Copyright 2021, American Chemical Society.

EV‐based mRNA therapy has shown promising results in preclinical and clinical studies for the treatment of diseases such as breast cancer, ovarian cancer, colorectal cancer, lung cancer, and glioblastoma. HChrR6‐mRNA loaded in EVs was used against breast cancer cells, resulting in the complete inhibition of their activity [28]. In another work, the same method was utilized for cancer cells, and the difference was that HChrR6 converted prodrug CNOB into toxic drug MCHB, contributing to the apoptosis of cancer cells [119]. In addition, immune escape and drug resistance are unignored issues for breast cancer. Ligand‐dependent corepressor (LOCR), as a biomolecule mediating cell differentiation, modulated the tumor immune escape and played a significant role in the process of antigen processing. In the context of LOCR function, EV‐based LOCR mRNA therapy combined with PD‐L1 was used for breast cancer. As a result, longer survival and little lung metastasis were found in preclinical models [120]. Because of its highly metastatic, ovarian cancer has a poor prognosis and unfavorable survival. Akira et al. found that metastatic ovarian cancer‐derived EVs carrying MMP1 mRNA, which is a destructive molecule for the peritoneal mesothelium barrier and a poor prognosis, facilitated peritoneal dissemination. A target and premise about EVs connected with peritoneal dissemination were provided for ovarian cancer based on the above thoughts [121]. Owing to the ability to deliver mRNAs to the liver and the potential alteration of functions, including promoting invasion and metastasis, colorectal cancer‐derived EVs were used to package LAT1 and ASCT2 mRNA, which are related to poor prognosis and associated with liver or kidney cancer to clarify their tropism. The results revealed that LAT1 mRNA loading in EVs was easier to be internalized by recipient cells and indicated that LAT1 mRNA promoted cell migration and proliferation [122]. In addition, the delivery of IL‐12 mRNA via EVs enhanced lung cancer treatment by fostering immune memory and augmenting the production of effector cells, thereby ameliorating the symptoms associated with the disease [123] (Figure 7A,B) Utilizing these mRNA loaded in EVs may have a profound effect on the uptake of therapeutic mRNA and the metastasis of cancer. Notably, bacterial outer membrane vesicles have shown promise in enhancing the tumor microenvironment and exhibiting anti‐tumor properties. However, the investigation into their mRNA profiles warrants further exploration [124].

Specific targets, large yield, and higher transfection and encapsulation efficiency in EV‐based mRNA therapy for cancer were further enhanced by engineering EVs. EVs can penetrate physiological barriers, including the BBB, and have a homing effect because of the specific protein on its surface. However, their specific target ability is not efficient for precision mRNA delivery, and the extent of EV‐based mRNA target delivery and improved immunotherapy of tumors has not been fully evaluated. Because of the above issues, Dong et al. constructed a microfluidic electroporation system that not only overexpressed CD64 but also attracted more IFM‐γ mRNA. The protein CD64, in combination with both anti‐CD71 and anti‐PD‐L1, enhanced the targeting efficiency of EV‐based mRNA towards glioblastoma tumors. In addition, IFM‐γ mRNA regulated the microenvironment of tumors by upregulating MHC‐1 to improve cancer immunotherapy [125, 126]. Notably, EV‐based mRNA therapy largely depends on abundant mRNAs and sufficient EVs. Cellular nanoporation significantly increased the amounts of EVs containing transcribed mRNA by electrical stimulation. EVs loading mRNAs expressing PTEN protein also prolonged survival and inhibited tumor growth [4] (Figure 7C). In addition, the combination of nano‐and milli‐second pulses also significantly increased the yield of EVs by about a 45‐fold increase. In this method, short‐duration and high‐intensity nanosecond electrical pulses can temporarily increase the membrane permeability of intracellular organelles (such as endoplasmic reticulum, Golgi apparatus, mitochondria, etc.) without damaging the plasma membrane so that external plasmid DNA can enter the intracellular organelles. Long‐duration, low‐intensity millisecond electrical pulses can increase the permeability of the cell plasma membrane without affecting the cell activity so that the EVs in the cellular organelles can be secreted to the outside of the cell, thus increasing the production of EVs [48] (Figure 7D). Although the yields of EVs loading mRNAs were alleviated to some extent based on the cellular nanoporation and the combination of nano‐and milli‐second pulses, transfection, and encapsulation efficiency limit its further application. The hybrid nanoparticles consisting of EVs and liposomes were constructed to improve the transfection and encapsulation efficiency. Compared with EV‐based mRNAs, hybrid nanoparticles showed higher transfection and encapsulation efficiency. For example, these hybrid nanoparticles‐based ALKBH5 mRNA, which correlates with N6‐methyladenosine modification and affects tumor progression, largely inhibited tumorigenesis and prolonged the survival of preclinical tumor models by restricting glycolysis [127]. Therefore, EVs, in combination with liposomes, may be a new choice for the high transfection and encapsulation efficiency of mRNA.

Gene therapy‐based EV loading mRNAs has also been applied in cancer fields. Chimeric antigen receptor (CAR) T‐cell therapy is popular in cancer because it allows modified T cells to completely kill cancer cells without the restriction of the histocompatibility complex [128]. However, the immunogenicity and risk of side effects of lentiviral transduction hamper its further development. Ke et al. utilized CRT mRNA in combination with EVs containing anti‐CD3/CD28 fragments, which interacted with CD3/CD28/TCR on the surface of T cells to activate T cells to accomplish CRT high expression in T cells and obtain a better effect on killing tumor cells [129]. Besides, suicide gene therapy is also a common gene therapy for cancer. Like CAR T therapy, unavoidable adverse effects and immunogenicity caused by conventional carriers are unignored issues. Therefore, uracil phosphoribosyl transferase (UPRT) suicide mRNA was loaded into non‐tumorigenic cells‐derived EVs to avoid adverse effects and immunogenicity. When these suicide mRNA‐based EV systems and 5‐FC were given to model mice and cells, glioblastoma tumor growth and cell viability were inhibited because UPRT converted non‐toxic 5‐FC to toxic 5‐UC, blocking tumor DNA and protein synthesis and further triggering tumor cells apoptosis [94].

### 6.2 EV‐Based mRNA for Tissue Regeneration

The application of EVs for tissue regeneration has garnered increasing attention due to their potential to facilitate recovery processes. However, the direct correlation between specific biomolecules, such as RNAs and proteins within these vesicles, and their beneficial effects on regeneration remains to be elucidated. Here, EV‐based mRNA regeneration therapy was summarized to indicate the relationship between mRNAs and tissue regeneration. Osteogenesis, angiogenesis, and collagen replacement mainly contribute to EV‐based mRNA regeneration (Table 2).

**TABLE 2:** EV‐based mRNA delivery for tissue regeneration.

mRNA	Administration	Diseases	Therapeutic outcomes	Ref
IL‐10 mRNA	Buccal injection	Bone generation	Activating cellular IL‐10/IL‐10R pathway. Osteoblasts were introduced, and bone resorption was also inhibited	[130]
TFAM mRNA	Intravenous injection	Bone generation	Mitochondrial oxidative phosphorylation (OXPHOS) and osteogenic differentiation	[131]
PI3K/AKT mRNAs	Not described	Angiogenesis	Activated PI3K/AKT and endothelial NOS pathways to promote angiogenesis	[132]
UBE2O mRNAs	Intravenous injection	Angiogenesis and wound repair	SMAD6 was restricted, and BMP2 was activated	[136]
collagen mRNA	Intravenous injection	Collagen regeneration	Restores collagen expression in photoaged skin and reduces the number and area of wrinkles	[137]

**FIGURE 8:**
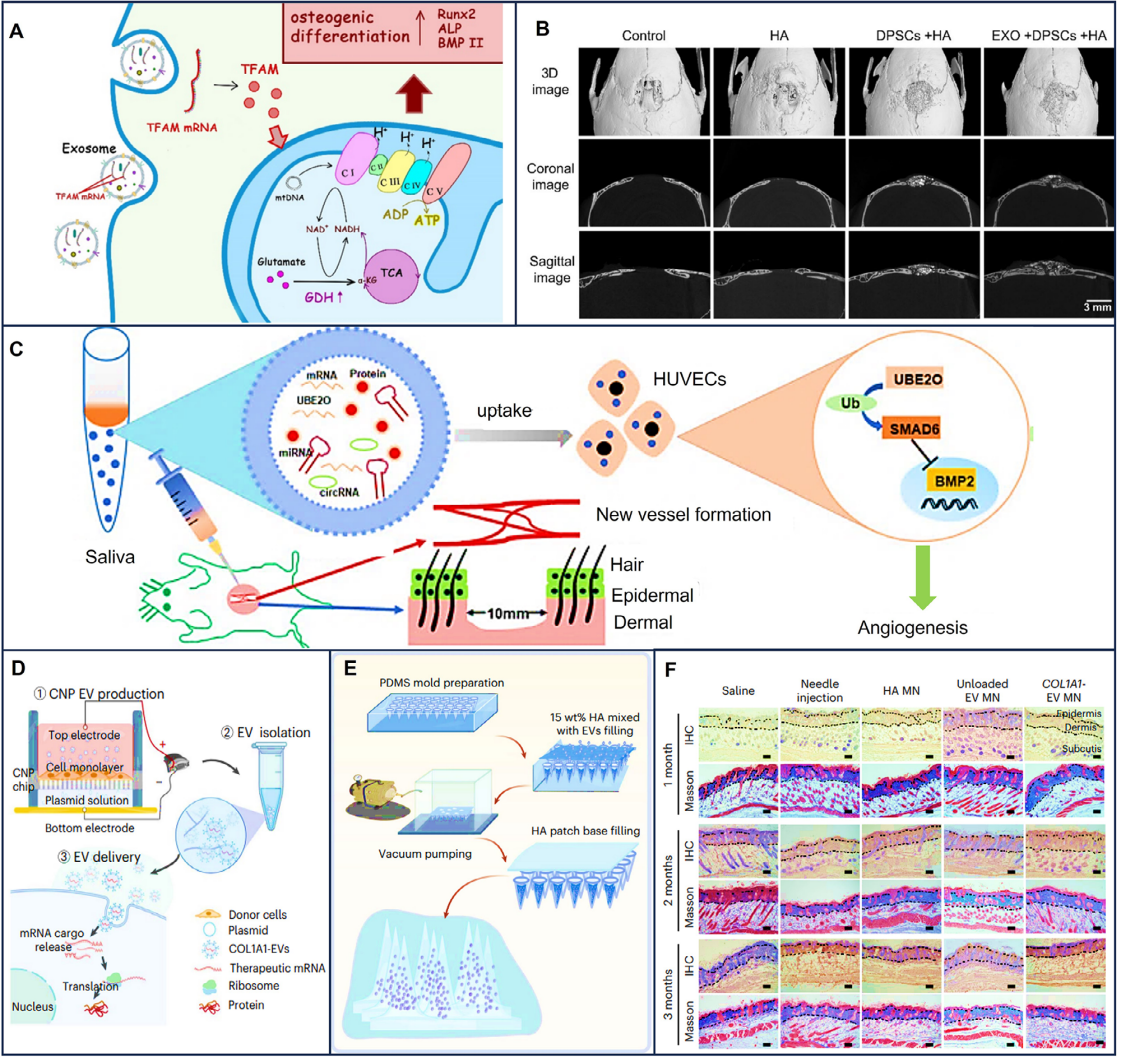
EV‐based mRNAs for osteogenesis and regeneration, wound repair, and collagen replacement. (A) TFAM mRNA shuttled by EVs (exosomes) promotes osteogenic properties of DPSCs through mitochondrial oxidative phosphorylation (OXPHOS) activation. (B) EVs enhance dental pulp stem cells‐mediated repair of the cranial bone defect. Reproduced with permission [131]. Copyright 2023, C Wiley‐Blackwell. Schematic illustration of (C) EV‐based UBE2O mRNA for wound repair. Reproduced with permission [133]. Copyright 2020, Springer Nature. (D) Cellular nanoporation (CNP)‐generated EVs are used for targeted nucleic acid delivery. (E)Microneedle fabrication. (F) EVs containing COL1A1 mRNA are used for collagen replacement. Reproduced with permission [10]. Copyright 2023, Springer Nature.

Osteoblasts and osteoclasts are two types of bone cells, and they maintain a dynamic balance to keep bones stiff and stretching. Chen et al. indicated that M2 macrophages participate in the progression of osteogenesis. They thus harnessed EVs from M2 macrophages encapsulating IL‐10 mRNA to trigger the IL‐10/IL‐10R signaling pathway, thereby promoting osteogenesis. Not only osteoblasts were induced but also osteoclasts were inhibited under the influence of M2 macrophages EVs loading IL‐10 mRNA. Bone resorption was also inhibited in this progress [130]. Apart from the cellular IL‐10/IL‐10R pathway, mitochondrial oxidative phosphorylation represents another significant avenue for osteogenesis. EVs delivering mitochondrial transcription factor A (TFAM) mRNA to dental pulp stem cells (DPSCs) elevated TFAM expression, thereby enhancing mitochondrial oxidative phosphorylation (OXPHOS) and osteogenic differentiation of DPSCs [131] (Figure 8A, B).

There are numerous examples of EVs functionally delivering mRNAs applied in cutaneous regeneration by angiogenesis. Angiogenesis, vital for cutaneous regeneration, benefits from the functional delivery of mRNA by saliva‐derived EVs. Mesenchymal stromal cells (MSCs)‐derived EVs are instrumental in cutaneous wound healing by engaging various components, including epidermal and dermal cells, growth factors, and blood vessels. EVs derived from MSCs loading mRNAs activated PI3K/AKT and endothelial NOS pathways, fostering angiogenesis [132]. In addition, saliva may also be a promising source of EVs involving regeneration because of the existence of many growth factors. Saliva EVs contain large amounts of ubiquitin‐conjugating enzyme E2O (UBE2O), and EVs loading UBE2O mRNA were transported into human umbilical vein endothelial cells (HUVECs) whose proliferation was restricted by SMAD family member 6 (SMAD6). As a result, SMAD6 was restricted, and growth factor‐like bone morphogenetic protein 2 (BMP2) was activated, thereby contributing to angiogenesis and wound repair [133] (Figure 8C). EV mRNAs were also applied in collagen‐replacement therapy. EVs containing collagen mRNA, when injected into the skin of mice, translated into protein within 12 h post‐injection, with expression peaking at 4 days and persisting for several weeks. Compared to LNPs‐mRNA, EVs with collagen mRNA not only restored collagen levels in photoaged skin but also reduced the number and area of wrinkles without causing local inflammation, unlike LNPs. In conclusion, these results underscore the promising potential of EVs loaded with mRNAs for collagen replacement and broader regenerative applications [10] (Figure 8D–F). Furthermore, a corresponding study has utilized EVs to simultaneously deliver mRNAs for both angiogenesis and osteogenesis in tissue engineering scaffolds. By using customized PEGylated polyglyceryl sebacate acrylate (PEGS‐A) hydrogels, therapeutic EVs (t‐EVs) carrying human vascular endothelial growth factor A (VEGF‐A) and bone morphogenetic protein 2 (BMP‐2) mRNAs were delivered to critical‐sized defects in rat femurs. The large‐scale production of EVs enriched with therapeutic mRNAs was achieved via a track‐etched membrane (TM‐nanoEP) cell nano‐electroporation system. Results demonstrated that localized and controlled release of t‐EVs significantly enhanced bone regeneration while reducing accumulation in other organs [134].

### 6.3 EV‐Based mRNA Therapeutics for Treatment of Other Diseases

#### 6.3.1 Neurodegenerative Diseases

EV‐mediated mRNA therapy offers significant advantages in brain disease treatment, including the facilitation of highly personalized therapies through precise gene modulation, minimal immunogenicity, the ability to traverse the BBB, the potential for early‐stage diagnosis and disease monitoring, and the circumvention of drug metabolism challenges. This avenue holds substantial promise for both research and therapeutic applications in brain therapy. However, it is imperative to acknowledge and address pertinent challenges, including enhancing delivery efficiency, ensuring safety, and ascertaining long‐term therapeutic efficacy. Currently, EV‐based mRNA therapy for brain diseases is primarily explored in conditions such as Parkinson's, Alzheimer's (Table 3).

**TABLE 3:** EV‐based mRNA delivery for treatment of neurodegenerative, infectious, hematological, vascular, and obesity‐related diseases.

mRNAs	Administration	Diseases	Therapeutic outcomes	Ref.
IL‐6, TNF‐α, IL‐6 mRNAs et al.	Not described	Alzheimer diseases	Expression differences in old and young samples	[135]
Catalase mRNA	Transcranial administration	Parkinson	Alleviated neuroinflammation	[88]
CUEDC2 mRNA	Not described	Amyotrophic lateral sclerosis	Biomarkers for amyotrophic lateral sclerosis	[164]
ZPAMt mRNA	Intravenous injection	HIV‐1	“Block and lock” the expression of HIV‐1	[138]
CRISPR‐Cas9 mRNA	Intravenous injection	Leukemia	miR‐125a and miR‐125b expression were decreased	[26]
KGF mRNA	Intravenous injection	Acute lung injury	Restoring lung protein permeability and reducing inflammation	[139]
Bmp7 mRNA	Intravenous injection	Obesity	Induce omental adipose tissue Browning	[140]
IRSE‐IL‐10 mRNA	Intravenous injection	Atherosclerosis	Responsive to miR‐155 and alleviated atherosclerosis	[93]

**FIGURE 9:**
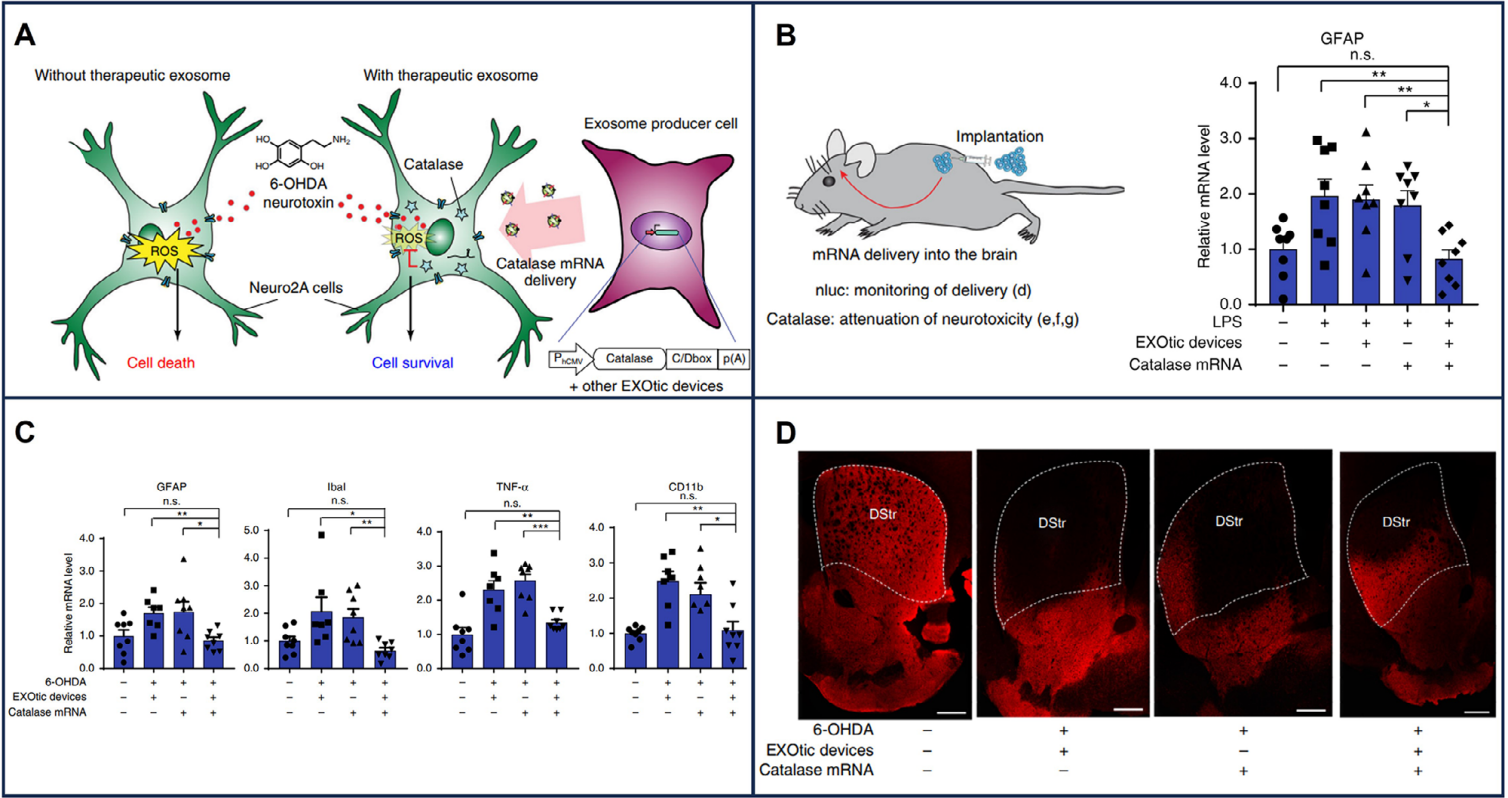
EV‐based catalase mRNA therapeutics for the treatment of Parkinson's disease. (A) EVs (exosomes)‐catalase mRNA protects the model of Parkinson's disease against neurotoxicity. (B,C) Therapeutic catalase mRNA was delivered using engineered EVs and inhibited neuroinflammation. (D) Immunostaining result of TH‐positive neurons. Reproduced with permission [88]. Copyright 2018, Springer Nature.

Significant distinctions in EV‐mRNA profiles between older and younger samples have been observed. For instance, macrophages stimulated with amyloid‐β_1‐42_ (Aβ) peptide and lipopolysaccharide (LPS) secreted increased levels of EVs carrying mRNA for IL‐6, TNF‐α, and IL‐12 in older samples compared to younger ones. Similarly, T cells stimulated with anti‐T‐cell antigen receptor antibodies produced a significant increase in EV‐mRNA in older samples. This difference in the expression of EV‐mRNA provided a meaningful context for AD‐associating mRNA [135]. Further, EV‐based mRNA therapies have been innovatively applied to brain diseases. For example, Ryosuke et al. constructed an EV production booster to produce enough EVs loading therapeutic mRNAs for Parkinson's disease. This production booster took HEK‐293T cells modified with STEAP3, syndecan‐4, a fragment of L‐aspartate oxidase, and a fragment of L‐aspartate oxidase to achieve high production of EVs as the main body of production. Notably, the above‐modified molecules are associated with EV biogenesis, the forming of MVB, and cellular metabolism. Next, potential cytosolic delivery helper connexin 43 enhances information transfer, and RVG‐Lamp2b, as a targeting module to target the brain, was constructed as an active packaging device for specific RNAs of EVs. Before these engineered cells were implanted in the brain, therapeutic catalase mRNA was specifically loaded into HEK‐293T‐EVs, leading to abundant EVs loading catalase mRNA secreted in the brain. These systems alleviated neuroinflammation induced by LPS injection or 6‐OHDA for the Parkinson model [88] (Figure 9) Given these examples, EVs carrying mRNAs play a crucial role in CNS communication, showcasing their potential in treating neurodegenerative diseases.

#### 6.3.2 Hematological Diseases

**FIGURE 10:**
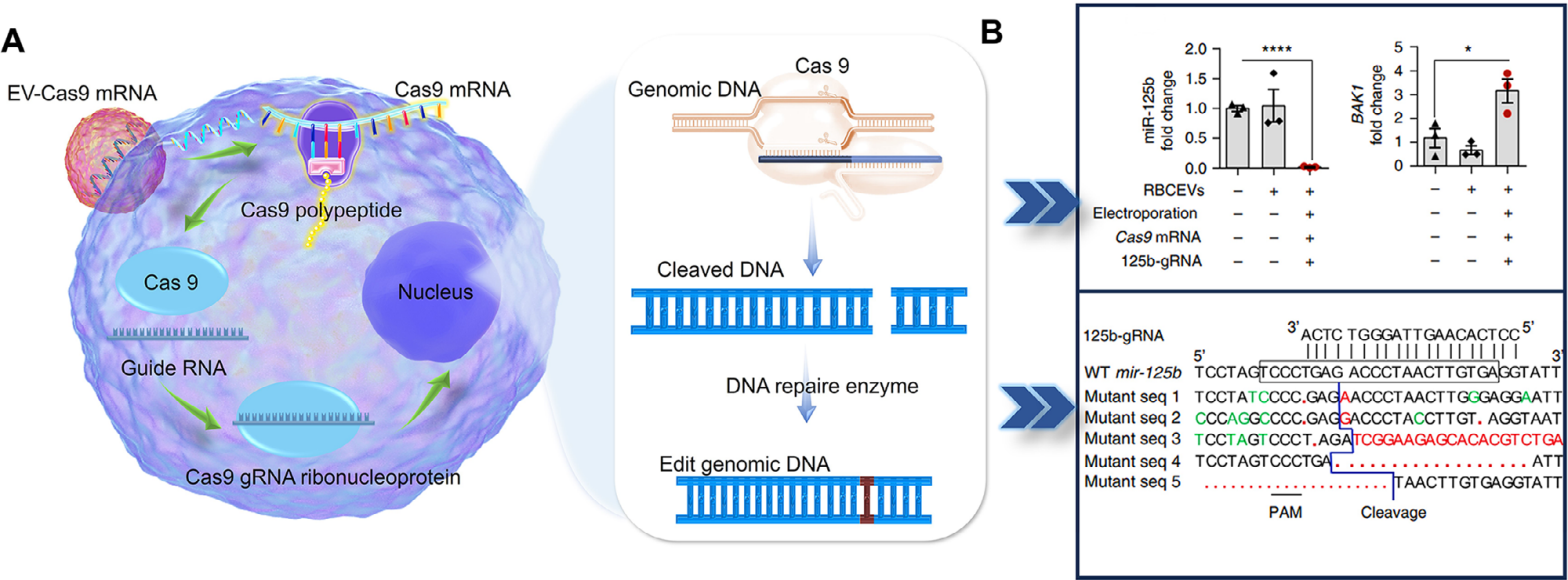
(A,B) EV‐based Cas9 mRNA mediated gene editing for the treatment of leukemia cells. EVs containing Cas9 mRNA are swallowed by target cells. Next, Cas9 mRNA is released and is translated into Cas protein. Cas9 protein is subsequently combined with the Guide RNA to form a Cas9 RNA ribonucleoprotein with a high affinity for DNA. This ribonucleoprotein connects with a DNA sequence complementary to guide RNA, meditating a double‐stranded DNA cut after it arrives in the nucleus. On the one hand, DNA break is then repaired by a mechanism called non‐homologous end joining, potentially leading to gene knockout. On the other hand, DNA break is replaced by a newly inserted DNA fragment in the presence of a repair template, which is called homologous directed repair. (C) As a result, a 98% reduction of miR‐125b expression and a 90% reduction of miR‐125a were realized in MOLM13 cells after a 2‐day treatment of EV‐based Cas9 mRNA.

EV‐based mRNA therapies have shown promising advancements in leukemia treatment. RBC EVs carrying Cas9 mRNA or gRNA targeting miR‐125b, a prevalent oncogenic microRNA in leukemia, significantly reduced its expression. As a result, 98% of miR‐125b expression and 90% of miR‐125a expression were decreased. This effective knockdown indicated that RBC‐EVs loading CRISPR‐Cas9 mRNA may be a new choice for leukemia treatment [26] (Figure 10). This approach highlights the efficacy of EV‐based mRNA therapy in gene knockdown or suppression without adverse effects.

#### 6.3.3 Infectious Diseases

Human immunodeficiency virus (HIV‐1) targets and compromises the human immune system, primarily by infecting and destroying essential CD4+ T cells, crucial for a robust immune defense. Despite efforts, challenges such as the lack of an effective vaccine, drug resistance, uneven distribution, and adverse effects hinder progress in combating HIV‐1 [136, 137]. To address these issues, EVs carrying mRNAs encoding epigenetic inhibitors have been explored to suppress HIV‐1 expression (Table 3). DNA Methyl Transferase, fused with Zinc Finger Protein ZFP‐362b targeting HIV‐1 LTR site 362, aims to reduce HIV transcription. Yet, achieving specific targeting, navigating to HIV‐1 affected areas, and sustaining anti‐HIV activity poses significant challenges. Utilizing EVs to deliver ZPAMt (ZFP fused to PWWP, ADD, and methyltransferase) mRNA promises to “block and lock” HIV‐1 expression, repressing virus expression and curtailing HIV progression [95][138].

#### 6.3.4 Lung Injury

EV‐based RNA therapy has also demonstrated potential in treating acute lung injury (ALI) (Table 3). Because of the known function of lung protein restoration and inflammation reduction with mesenchymal stem cells (MSC), MSC‐EVs were utilized to package keratinocyte growth factor (KGF), mediating the restoration of MSC in keratinocyte growth factor to treat and restore acute lung injury. Results indicated that MSC‐EVs restored lung protein permeability over 24 h, while MSC‐EVs loading KGF mRNA significantly restored ALI by restoring lung protein permeability and reducing inflammation [139].

#### 6.3.5 Atherosclerosis

Atherosclerosis (AS) treatment also benefits from EV‐based mRNA therapy. The proposed engineered IRSE‐IL‐10 mRNA was loaded into EVs, responsive to the miR‐155 increase in AS. Notably, IRSE‐IL‐10 mRNA translation could be specifically activated in atherosclerosis plaque, while other regions without inflammation have little translation because of the absence of miR‐155. This specific activation originated from the alternation of IRES, whose structure of translation inhibition was converted to translation activation, contributing to a combination of ribosome and RNA. As a result, EVs loading IRSE‐IL‐10 mRNA were specifically delivered to atherosclerosis plaque, and IL‐10 protein expressing further alleviated atherosclerosis [93].

#### 6.3.6 Obesity Disease

**FIGURE 11:**
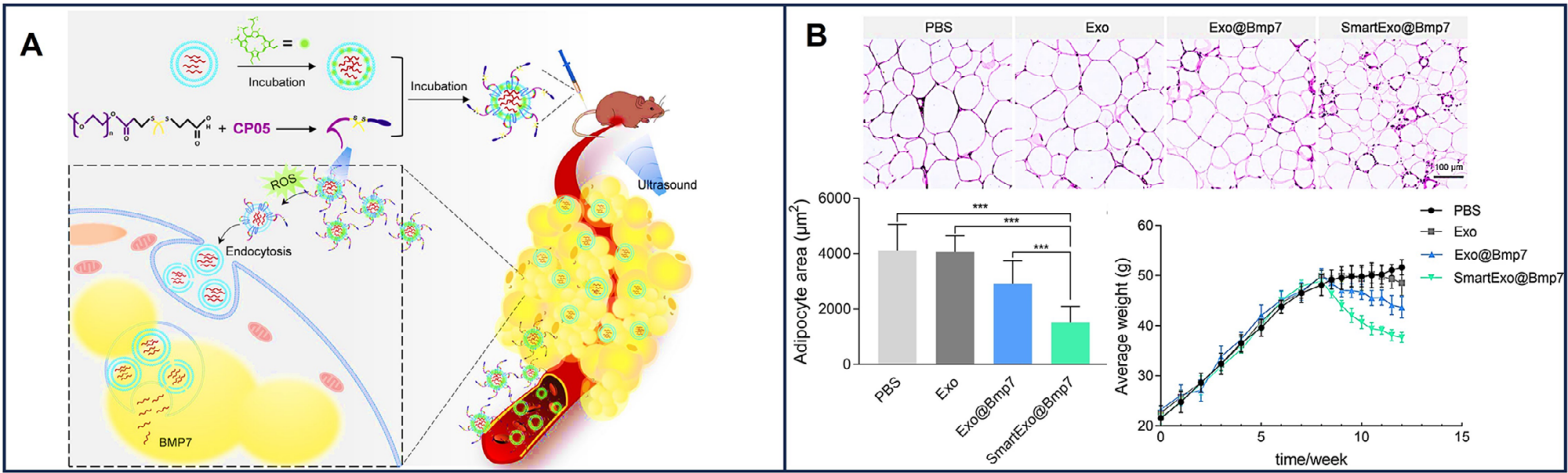
EV‐based mRNA therapy obesity treatment. (A) White fat browning is induced by EVs containing Bmp7 mRNA. (B) H.E. and relative area of lipid droplets for omental adipose tissue. SmartExo@Bmp7 significantly reduced the weight of mice. Reproduced with permission [140]. Copyright 2021, Springer Nature.

In one study aimed at combating obesity, a targeted mRNA platform enhanced endogenous circulation. This platform combined CP05‐thioketal (TK)‐mPEG with CD63 on EV surfaces and mPEG for phagocytosis protection, effectively delivered Bmp7 mRNA to omental adipose tissue (OAT) in mice, inducing OAT browning with ultrasound assistance [140] (Figure 11A, B). In summary, these examples show the significant potential of EV‐based mRNA therapy in targeting, inflammation reduction, and gene editing for various diseases.

## 7 EV‐Based mRNA Vaccines

**FIGURE 12:**
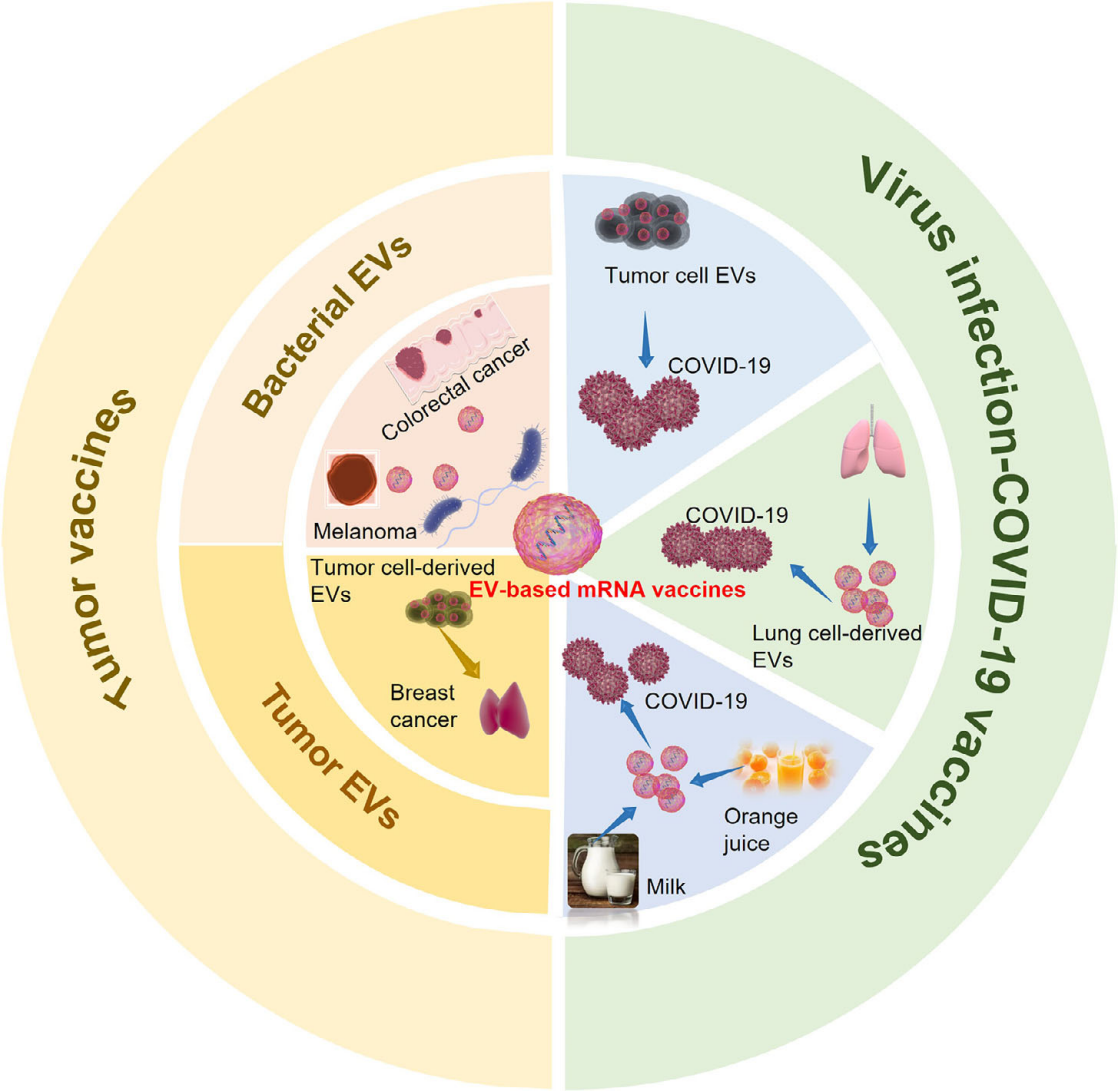
Schematic illustration of EV‐based mRNA vaccines.

mRNA vaccines may represent an advanced alternative to conventional vaccines [112, 141, 142]. Once the mRNA sequence is identified, the specific vaccine will be immediately made. Changeability in specific sequences makes them adaptable to emerging pathogens and various pathogens. In addition, both B and T cells are activated when mRNA‐based vaccines induce cellular and humoral immunity, leading to the enhancement of immunity. The risk of infection is significantly decreased because mRNA vaccines cannot replicate, thus having little opportunity to become its pathogenic form. However, the instability, including clearance by the immune system and the necessity of low‐temperature storage, limits its clinical application [67]. Sufficient mRNA translation in interest cells is required in clinical applications, and internalization or translation of mRNAs is limited by larger and negatively charged mRNA or type I interferons suppressing translation initiator [68, 143]. Furthermore, insufficient targeting may be an unignored issue for mRNA vaccines. EVs, as endogenous vesicles, are not eliminated by the immune system, and mRNA stability is thus improved when it is encapsulated in EVs. Meanwhile, EVs containing mRNAs can be internalized into cells by fusion or phagocytosis, facilitating mRNA entry into cells. The specific protein within EVs may be combined with specific receptors on the surface of target cells. Upon these characteristics of EVs, various problematic issues of mRNA vaccines were solved [49, 113, 141, 144] (Figure 12).

### 7.1 Mechanism of EV‐Based mRNA Vaccines

**FIGURE 13:**
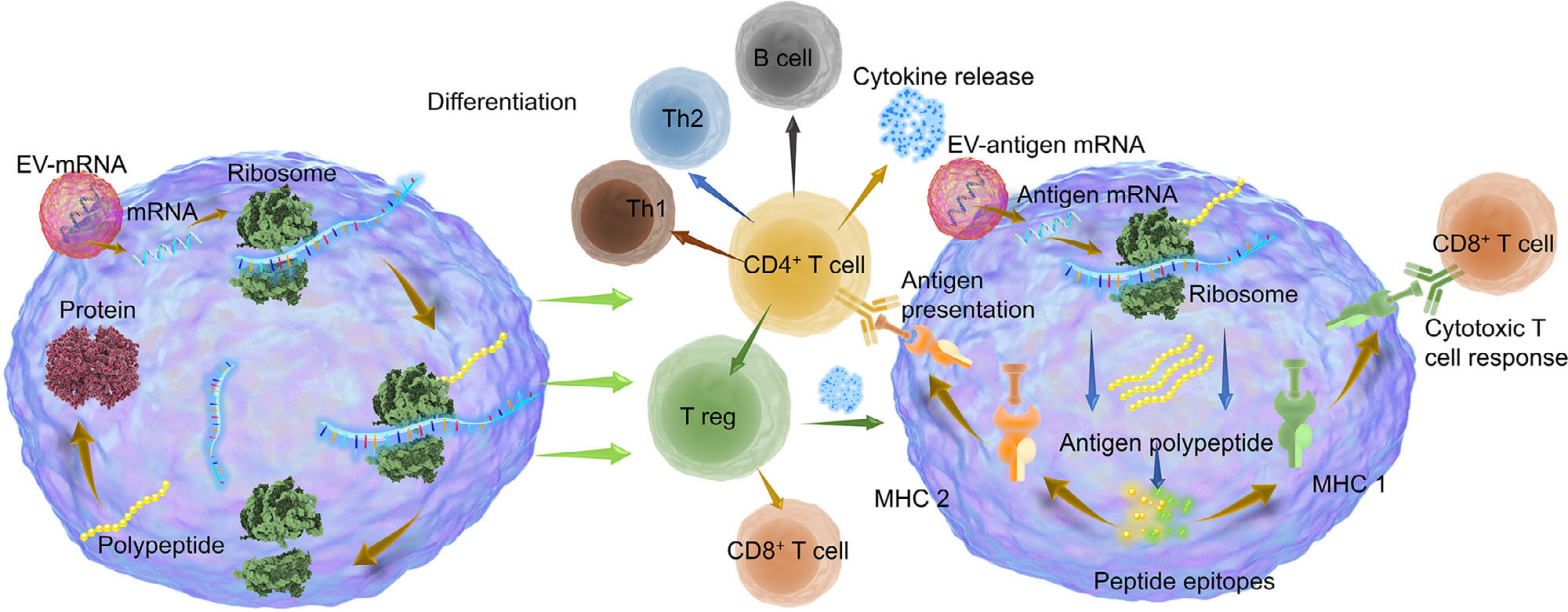
EV‐based mRNA translation and vaccine function. mRNA is recognized and connected with ribosomes after EVs loading mRNAs enter the cell. The ribosome scans the mRNAs and begins translation when it recognizes the start codon. The process of translation is terminated until the appearance of a stop codon, resulting in the release of polypeptides. These polypeptides are generally modified and become proteins with specific functions. However, these peptides are called antigen peptides and are processed into small peptide epitopes in antigen‐presenting cells. Notably, these small peptide epitopes could bind to MHC‐1 and MHC‐2, and these peptide epitopes will be transferred to the cell surface, followed by MHC‐1 and MHC‐2. As a result, they were separately presented to CD4+ T cells and CD8+ T cells, leading to the activation of cellular and humoral immunity.

By virtue of EVs, mRNAs could be successfully delivered to the antigen‐presenting cell (APC) despite their larger molecular size and negative charge. mRNAs, in combination with ribosome and tRNA, form an initiation complex that ensures precision and rapid translation after mRNAs enter the cytosol. Subsequently, the corresponding amino acid carried by tRNA is paired with mRNA codons while ribosomes move along the mRNAs and polypeptides, resulting in polypeptide synthesis. Next, newly produced proteins were cleaved into small fragments, resulting in the formation of antigen peptides in the endoplasmic reticulum. Antigen peptides are then combined with the MHC complex to activate T cells in two ways. On the one hand, the antigenic peptide is transported from the cytoplasm to the endoplasmic reticulum by the transporter associated with antigen processing (TAP). After this transport, the antigenic peptide combined with MHC‐1 forms the MHC‐I antigenic peptide complex, which is presented on the surface of APC cells. Finally, the CD8 cell receptors combine with this MHC‐1 antigenic peptide complex to activate CD8 T cells. Notably, co‐stimulatory molecules, including CD28 and B7, also mediate this activated progress. On the other hand, some antigenic proteins exist in endosomes, which are degraded by endosomes to produce antigenic peptides, which then bind to MHC‐2 to form the MHC‐2 antigenic peptide. Similarly, this type of antigenic peptide is also presented on the surface of APC and next binds to the receptors of CD4+ cells. As a result, CD4+ T cells are activated. In conclusion, the EV‐based mRNA vaccine activates both cellular and humoral immunity [145, 146] (Figure 13).

### 7.2 EV‐Based mRNA Vaccines Against Infectious Diseases

**FIGURE 14:**
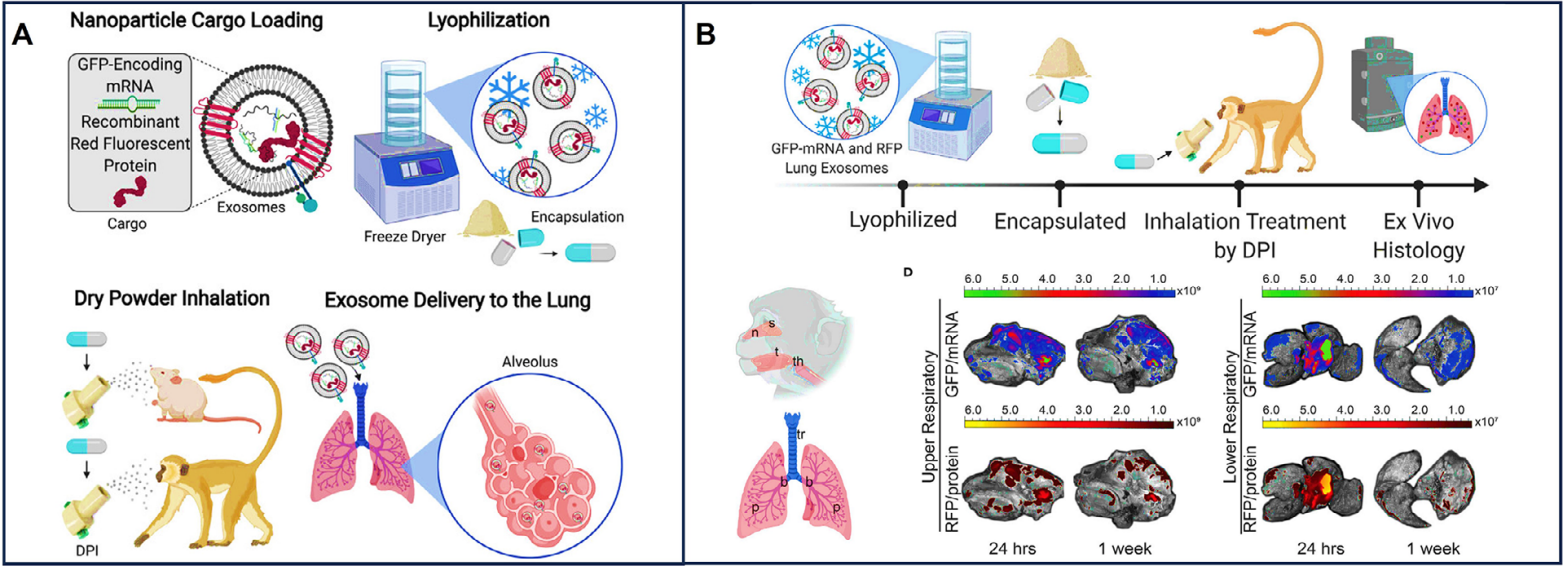
EV‐based mRNA vaccine for the treatment of infectious diseases. (A) Inhalable dry powder mRNA vaccines based on EVs for SARS‐CoV‐2. (B) Distribution of lung‐derived EVs vaccine via dry powder inhalation in African green monkeys. Reproduced with permission [21]. Copyright 2022, Cell Press.

Despite EV‐based mRNA vaccines being used in clinics less at present, their efficient treatment has been further verified in SARS‐CoV‐2 and tumor treatment (Table 4). Based on various specific requirements originating from tumor or infection treatment, different origins of EVs, including bacteria, milk, and orange juice‐derived EVs, have been applied for delivering mRNA vaccines to obtain greater immune activation, higher yield, and more convenient application. EV‐based mRNA vaccine is most used in SARS‐CoV‐2 due to the outbreak of COVID‐19. EVs are originally a substitute for LNPs to deliver mRNAs. Related studies revealed that EVs containing mRNA vaccines elicited little cellular toxicity; meanwhile, they produced few side effects in vitro or in vivo. Besides, luciferase expression lasted for 70 days in mice injected repeatedly six times with EV‐based Antares2 mRNA, demonstrating sustained protein expression with fluorescence. EV‐based functional mRNA was further applied in vivo to verify its vaccine efficacy after confirming the safety and long‐term expression of the EV‐based mRNA vaccine. mRNAs encoding the spike (S1) protein of SARS‐CoV‐2 and fusion protein containing nucleocapsid protein, membrane protein, envelope protein, fragments of the spike, as well as lamp1 protein (LSNME) were packaged into 293T‐derived EVs, and this system was injected to C57 mice. As a result, this EV‐based mRNA vaccine induced the production of antibody‐S1 and antibody‐N while significantly activating CD3 and CD8 T cell proliferation. Notably, the minimal side effects of the EV‐based mRNA vaccine in mice were also verified by consistent body weight, white blood cell profiles, and organ pathology with normal mice [99]. As we all know, COVID‐19 is a respiratory disease directly related to the lungs. However, conventional mRNA vaccines face challenges reaching the bronchioles and lung parenchyma due to complex defense mechanisms, such as surfactants that prevent particulates and microbes from entering the lungs [147, 148]. Depending on these biological characteristics of the lung, the lung‐derived EVs with high lung histocompatibility may be crucial in increasing the lung target efficiency of the mRNA vaccine. Kristen et al. utilized lung‐derived EVs to load mRNAs encoding S protein (S‐Exo) according to the high lung histocompatibility of lung EVs. Meanwhile, because of the instability of EV‐based mRNA, this system was further processed into an inhalable dry powder, resulting in one month of storage with retained function. Compared with lipid‐containing mRNA encoding S protein (S‐Lip), S‐Exo exhibited greater biodistribution and longer retention time in both mouse and African green monkey lungs, as lung‐derived EVs possess a parent‐cell signature that helps avoid lung mucoadhesion. Based on this enhanced distribution, S‐Exo demonstrated a more rapid virus clearance than S‐Lip [21] (Figure 14A,B).

**TABLE 4:** Summary of EV‐based mRNA vaccines.

Target mRNAs	Route of administration	Diseases or infections	Therapeutic outcomes	Ref.
S1and LSNME mRNA	Intravenous injection	SARS‐CoV‐2	Inducing antibody‐S1 and antibody‐N production and CD3, CD4+, and CD8+ T cell proliferation are significantly activated.	[25]
S mRNA	Suction administration	SARS‐CoV‐2	Showing more biodistribution, more rapid clear virus, and longer retention time in the lung.	[21]
RBD mRNA	Oral administration	SARS‐CoV‐2	Activating mucosal immune and inducing T cell proliferation.	[151]
RBD mRNAs	Oral administration	SARS‐CoV‐2	Neutralizing antibodies in mice and RBD peptides were successfully induced.	[23]
L7Ae mRNA	Oral administration	Melanoma and colon cancer	Rapid mRNA loading, endosomal escape, and inhibition of tumor growth	[82]
GM‐CSF mRNA	Intradermal administration	Breast cancer	Induced cancer cell apoptosis, prevented tumor growth, promoted DC maturation, and greatly elicited T cell immune response	[156]
IL‐12 mRNA	Inhalation administration	Lung cancer	Promoted systemic immunity and was used for the treatment of lung cancer	[123]

Although animal cell‐derived EV‐based mRNA vaccines have been successful in SARS‐CoV‐2, challenges such as EV yield and the inconvenience of intermuscular injection during the COVID‐19 outbreak have hampered further development. Plant EVs, like orange juice‐derived EVs, are also utilized as delivery platforms for mRNA vaccines to treat SARS‐CoV‐2. Based on their convenient source and the resistance to acid or basic environment leading to good gastrointestinal stability, not only EV yield is solved, but also a great platform of mRNAs oral delivery is constructed [149, 150]. Therefore, orange juice‐derived EVs packaged mRNA, encoding S, Spike RBD (S1), and nucleocapsid phosphor (N) protein of SARS‐CoV‐2, as oral and intranasal vaccine of COVID‐19. Apart from the conventional enhancement of stability and translation of functional mRNAs, effective activation of lymphocytes was achieved, as evidenced by CD4+ T cell proliferation and the increased expression of activation markers, including CD25, CD69, and HLADR. In addition, IgA antibodies were generated following oral and intranasal administration, indicating that mucosal immune responses were specifically activated by these routes compared to intramuscular administration. Furthermore, orange juice EVs containing SARS‐CoV‐2 mRNA administered orally and intranasally elicited a stronger humoral immune response with specific IgM and IgG and induced a robust T‐cell response. Therefore, plant‐derived EVs may be an efficient delivery platform for mRNA vaccines, especially in activating mucosal immunity [151]. The milk‐derived EV mirrors the high yield of the orange juice‐derived EV but is primarily used as an oral mRNA vaccine. Zhang et al. suggested that milk‐derived EVs could overcome the complex gastrointestinal microenvironment while penetrating the gastrointestinal barrier to activate immune cells in the gastrointestinal tract [152]. Therefore, RBD mRNA encapsulated in milk‐derived EVs constituted a new oral vaccine for SARS‐CoV‐2, resulting in the successful induction of neutralizing antibodies and RBD peptides in mice. In conclusion, milk‐derived EVs also present a novel platform for oral mRNA vaccines [23].

### 7.3 EV‐Based mRNA Vaccines Against Tumors

**FIGURE 15:**
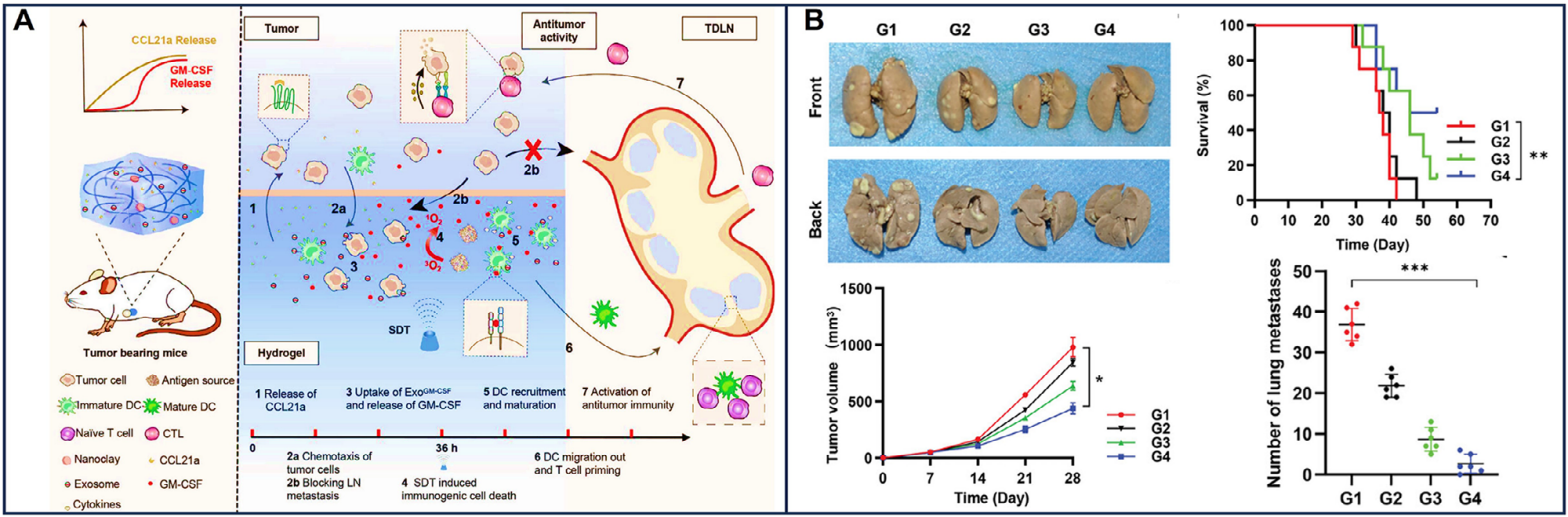
EV‐based mRNA vaccine for the treatment of tumors. (A) Modular hydrogel vaccine‐based EVs containing mRNAs. (B) Modular hydrogel vaccine inhibits lung metastasis, promotes survival and reduces the tumor volume of mice. Reproduced with permission [156]. Copyright 2023.

EV‐based mRNA vaccines are utilized not only in fighting infectious diseases but also in cancer treatment (Table 4). Currently, bacteria, tumor cells, and immune cell‐derived EVs have been employed to carry functional mRNAs for cancer vaccines. Bacteria‐derived EVs contained many pathogen‐associated molecular patterns that could further activate the immune response and promote efficient uptake by DCs. Based on this unique advantage, bacteria‐derived EVs could activate innate immunity without an immune adjuvant. In addition, rapid mRNA loading and endosomal escape are facilitated by linking EVs with RNA binding protein, L7Ae, and lysosomal escape protein, listeriolysin O. Because L7Ae, fused to the protein on the surface of EVs, could match the binding sequence added to mRNAs, allowing for quick mRNAs packaging. Following this, EVs containing mRNAs circumvent endosomal entrapment with the aid of listeriolysin O, which promotes the formation of pores in the endosomal membrane. Owing to these specific modifications in EVs, bacteria‐derived EVs loading therapeutic mRNA produced long‐term immune memory and significantly inhibited the growth of melanoma and colon cancer [82]. To further expand the vaccine's immune effect, complex materials with immune response and tumor cell killing have been incorporated into vaccine formulations. In one experiment, a novel strategy involving hydrogels containing EV‐based mRNA vaccines was proposed to recruit cancer cells and stimulate antigen presentation. CCL21, with the ability to promote tumor metastasis [153, 154] and tumor cells‐derived EVs, containing GM‐CSF mRNA with the antitumor ability [155] and sonosensitizer Chlorin e6 (Ce6), were added in hydrogels. CCL2 first recruited tumor cells to hydrogels, and then EVs loading GM‐CSF mRNA and Ce6 were swallowed by these tumor cells, resulting in tumor cells killed by sonodynamic therapy while DCs were stimulated to mature state by the GM‐CSF coordinating with dead tumor cells for further antitumor effect. As a result, these innovative hydrogels induced cancer cell apoptosis, hindered tumor growth, promoted DC maturation, and significantly boosted T cell immune response. [156] (Figure 15A,B). Furthermore, IL‐12 mRNA loaded by EVs (IL‐12‐Exo) derived HEK cells promoted systemic immunity and was used to treat lung cancer. Based on superior lung distribution and tumor specificity, IL‐12‐Exo effectively delayed tumor growth, while IL‐12‐exo induced IFNγ to regulate TME. In addition, IL‐12‐Exo treatment also initiated systemic antitumor T‐cell responses [123].

In conclusion, although not yet being widely utilized in a clinical setting, EV‐based mRNA vaccines have demonstrated significant advancements in treating both tumors and infections. Moreover, various sources of EVs, such as those derived from animal cells, plants, and bacteria, have enhanced therapeutic effects in terms of stability, targeting accuracy, biocompatibility, gastrointestinal stability for oral administration, and immune activation. Thus, EV‐based mRNA vaccines may represent a promising direction for future vaccine development.

## 8 Conclusion

mRNA has garnered significant attention in fields such as oncology and viral infections (particularly in the context of COVID‐19 vaccines) due to its efficient transcription, high safety profile, and ease of adjustment and production. However, mRNA is susceptible to degradation in vivo, necessitating delivery vehicles to maintain its stability and delivery efficiency. EVs, as natural EVs, possess numerous advantages for mRNA delivery. The lipid bilayer structure of EVs serves as the main protective barrier for mRNA, shielding it from degradation, while membrane proteins such as CD47 further enhance this protective environment by inhibiting immune cell activation and attack. Additionally, EVs possess targeting capabilities. Depending on the parental cells, EVs can carry mRNA to specific tissues or organs and physiological barriers, such as lung‐derived EVs exhibiting excellent lung distribution. Arc protein interacts with mRNA through ion interactions to facilitate mRNA transfer to multivesicular bodies (MVBs) [157], which are eventually secreted into the extracellular space to form EVs. Therefore, EVs possess inherent RNA carrier properties. EVs can also effectively deliver their cargo mRNA to target cells through cellular uptake mechanisms such as endocytosis or fusion. Based on the above advantages, EV‐based mRNA therapeutics and vaccines have shown considerable promise in numerous preclinical studies spanning various fields, including oncology, regenerative medicine, neurodegenerative disorders, infectious diseases, hematology, vascular pathology, and metabolic syndromes.

However, EV‐based mRNA therapeutics and vaccines have not yet progressed to clinical trials, due to the persistent challenge of enhancing the efficiency of mRNA encapsulation within EVs. To address these issues, alternative strategies beyond exogenous electroporation, ultrasound, incubation, and chemical reagents include engineering source cells to yield EVs enriched with RNA‐binding proteins or employing cellular electroporation for enhanced mRNA loading efficiency. Compared to exogenous methods, endogenous techniques such as engineering source cells offer the capability to achieve efficient mRNA loading into EVs without causing EV damage. For example, the efficient association loading of mRNA into EVs is achieved by hijacking mRNA‐binding proteins during the biogenesis of EVs. Besides, the continued progress of EV‐based mRNA therapeutics and vaccines is also hampered by other challenges, such as the scalable production of EVs and the maintenance of storage stability. Henceforth, in order to facilitate efficient clinical translation of EV‐based mRNA therapeutics and vaccines, future efforts should be dedicated to developing methodologies for mRNA loading, standardized EV characterization techniques, quality control, storage, and biomedical applications. Notably, although EV‐based mRNA therapeutics and vaccines have not yet entered clinical trials, both mRNA and EVs have made some significant strides in clinical experimentation. For instance, mRNA‐based protein therapies have advanced to clinical stages. The intracellular protein expression by mRNA‐3927 as a protein replacement therapy has demonstrated a therapeutic efficacy of 70% in patients with rare diseases [158]. Furthermore, engineered EVs candidate drugs have entered phase I clinical trials as of 2020 [35]. Therefore, EV‐based mRNA therapeutics and vaccines have very potential, and this technology may revolutionize the delivery of next‐generation mRNA treatments, vaccinations, cancer immunotherapy, and personalized treatments.

In conclusion, EV‐based mRNA holds immense promise for the therapy and vaccine fields. EV‐based mRNA therapy and vaccines may further benefit from strong multidisciplinary collaborations, such as the combination with genetic engineering or multifunctional biomaterial engineering. To advance mRNA‐EV therapies, it is crucial to rigorously test and address challenges related to safety, efficacy, and manufacturing standards. As EV technology continuously evolves, establishing standardized production and quality control processes, along with formulating corresponding regulatory standards and guidelines, is essential for the standardized development of EVs. The recently released MISEV2023 guidelines offer valuable insights into in vivo methods for studying EVs, which can help design preclinical and clinical studies of mRNA‐EVs, focusing on understanding their biodistribution and therapeutic potential. Considering the tremendous potential and recent expansion of the field, EV‐based mRNA therapy is expected to have a high level of success in the future.

## Conflicts of Interest

The authors declare no conflicts of interest. Yuanyu Huang is a member of the *Exploration* editorial board, and he was not involved in the handling or peer review process of this manuscript.
